# Potential of rice landraces with strong culms as genetic resources for improving lodging resistance against super typhoons

**DOI:** 10.1038/s41598-021-95268-0

**Published:** 2021-08-04

**Authors:** Tomohiro Nomura, Yoshiaki Seki, Makoto Matsuoka, Kenji Yano, Koki Chigira, Shunsuke Adachi, Francisco J. Piñera-Chavez, Matthew Reynolds, Satoshi Ohkubo, Taiichiro Ookawa

**Affiliations:** 1 Graduate School of Agriculture, Tokyo University of Agriculture and Technology, 3-5-8 Saiwai-cho, Fuchu, Tokyo 183-8509 Japan; 2 Bioscience and Biotechnology Center, Nagoya University, Furo-cho, Chikusa, Nagoya 464-8601 Japan; 3 Statistical Genetics Team, RIKEN Center for Advanced Intelligence Project, Nihonbashi 1-chome Mitsui Building, 15th floor, 1-4-1 Nihonbashi, Chuo-ku, Tokyo 103-0027 Japan; 4 College of Agriculture, Ibaraki University, 3-21-1 Chuo, Ami, Inashiki, Ibaraki 300-0393 Japan; 5 Global Wheat Program, International Maize and Wheat Improvement Center (CIMMYT), Texcoco, Mexico 56237 Mexico

**Keywords:** Genetics, Plant sciences

## Abstract

It is generally believed that rice landraces with long culms are susceptible to lodging, and have not been utilized for breeding to improve lodging resistance. However, little is known about the structural culm strength of landraces and their beneficial genetic loci. Therefore, in this study, genome-wide association studies (GWAS) were performed using a rice population panel including Japanese rice landraces to identify beneficial loci associated with strong culms. As a result, the landraces were found to have higher structural culm strength and greater diversity than the breeding varieties. Genetic loci associated with strong culms were identified, and it was demonstrated that haplotypes with positive effects of those loci were present in a high proportion of these landraces. These results indicated that the utilization of the strong culm-associated loci present in Japanese rice landraces may further improve the lodging resistance of modern breeding varieties that have relied on semi-dwarfism.

## Introduction

Increasing grain yield is important to meet the global demand for major crops, which is expected to increase at least until 2050^1–3^. In the 1960s, as a result of *semidwarf 1* (*sd1*) being introduced into rice, plant height was reduced, and since then, lodging-resistant varieties have been developed and are widely used. This event averted a food security crisis and along with similar interventions in wheat and other cereals has been recognized as the “Green Revolution”^4–6^. However, the short stature promoted by *sd1* in improved rice varieties, is not enough to avoid lodging especially under strong typhoons that will further increase intensity in East and Southeast Asia^7, 8^. It has also been suggested that semi-dwarf varieties have reduced culm strength and plant biomass compared to the original variety^7, 9^. Therefore, it is important to aim for high yields by increasing lodging resistance provided by increased culm strength instead of relying solely on culm dwarfism in the future.

There are two types of lodging resistance in transplanting rice cultivation: bending-type and breaking-type lodging^10^. Breaking-type lodging can cause the panicles to fall to the surface, making harvesting difficult^11^ and causing viviparous germination of grains soaked in water that will reduce the grain eating quality^12^. Therefore, enhancing breaking-type lodging resistance is an important issue for crop production. The bending moment at breaking (BM) of basal culm is an index of breaking-type lodging resistance^13^. It is composed of section modulus, which in turn is affected by the diameter and thickness of a culm, and bending stress, which is affected by the chemical composition such as cellulose and lignin and their densities in culm cell wall^14, 15^.

Identification and pyramiding of quantitative trait loci (QTLs) associated with strong culms in addition to *sd1* constitute an important approach to breeding rice varieties with high breaking strength to withstand typhoons of increasing intensity. In our previous studies, superior alleles of *STRONG CULM 1* and *2* (*SCM1* and *SCM2*) QTLs, which increase culm strength, were detected in an *indica* variety Habataki^16^, similarly *STRONG CULM 3* and *4* (*SCM3* and *SCM4*) QTLs were detected in a tropical *japonica* variety Chugoku 117^17^, and the genes responsible for *SCM2* and *SCM3* were identified as *APO1 ABERRANT PANICLE ORGANIZATION 1* (*APO1*)^16, 18^ and *FINE CULM 1* (*FC1*)^17, 19, 20^, respectively. In addition, many other QTLs associated with strong culms have been identified^21–28^.

It has been reported that, *japonica* landrace varieties in Japan have more agriculturally useful traits when compared with *japonica* breeding varieties. For instance, under phosphorus-limiting conditions, the *japonica* landrace Akamai developed an extensive root system compared with that of the *japonica* breeding variety Koshihikari to obtain more phosphorus and efficiently redistributed it within the plant^29^. However, few studies have comprehensively compared various varieties for culm-associated traits among Japanese genetic resources^13, 28^.

Therefore, we hypothesize that there are novel QTLs that could enhance culm strength in *japonica* varieties cultivated in Japan, including landrace varieties that have not been used for breeding yet. To date, the progeny of crosses between two parents have often been used as materials for analysing and identifying QTLs^30–32^. However, this method of analysis has the disadvantages of requiring cross-breeding to grow the materials, limiting the number of varieties that can be analysed at one time. On the other hand, the development of next-generation sequencing has recently enabled the use of GWAS, which have been shown to be powerful tools for solving the abovementioned problems, as these studies allow comprehensive analysis of varieties without the need for crossing^33–36^. Therefore, to test our hypothesis, we used GWAS to search for strong culm-associated QTLs in Japanese *japonica* varieties, including landraces.

Thus, in the current study, we examined the phenotypic characteristics of lodging resistance associated traits in Japanese rice varieties and novel QTLs associated with strong culms from *japonica* varieties using GWAS. Furthermore, we investigated the proportions of the superior alleles between landraces and breeding varieties.

## Results

### Phenotypic analysis of traits associated with lodging resistance in Japanese rice

**Figure 1:**
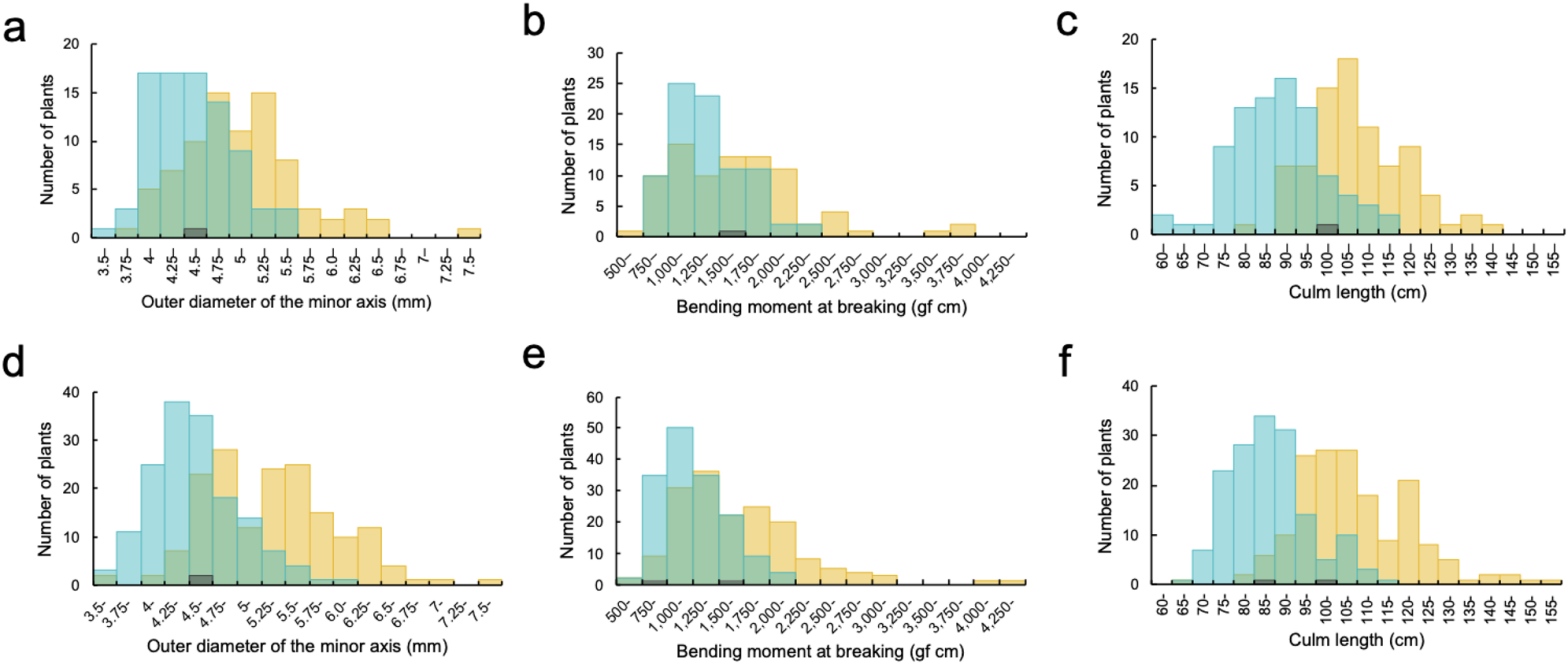
Frequency distribution of lodging resistance-associated traits. Histograms of phenotypic values: (**a**) ODMI in 2018; (**b**) BM in 2018; (**c**) culm length in 2018; (**d**) ODMI in 2019; (**e**) BM in 2019; (**f**) culm length in 2019. The yellow, blue and black bars indicate landraces, breeding varieties and unidentified varieties, respectively.

**Figure 2:**
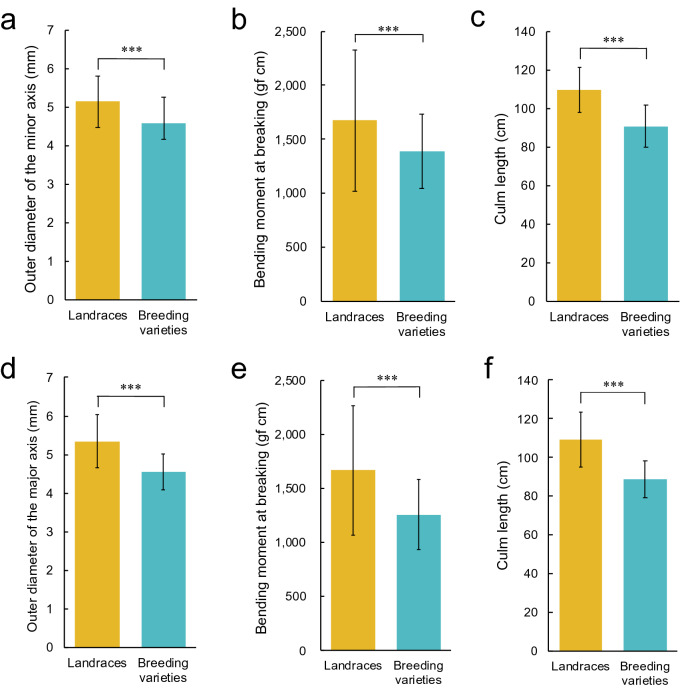
Trait values of landraces vs. breeding varieties. Bar graphs of mean (SD) phenotypic values: (**a)** ODMI in 2018; (**b)** BM in 2018; (**c)** culm length in 2018; (**d)** ODMI in 2019; (**e**) BM in 2019; (**f**) culm length in 2019. The yellow and blue bars indicate landraces and breeding varieties, respectively. *** indicates significant differences at *P* < 0.001 (two-tailed Welch’s t-test). Two-tailed Welch’s t-test was performed by R software (https://www.R-project.org/)^65^.

In GWAS, it is necessary that phenotypes in rice panels are highly diverse to detect QTLs efficiently^35^. Therefore, traits associated with lodging resistance were measured to evaluate the phenotypic diversity. Figure 1 shows the frequency distribution of phenotypes of Japanese rice, including both landraces (in yellow) and breeding varieties (in blue). For the outer diameter of the minor axis (ODMI), the landraces ranged from 3.78 to 7.68 mm in 2018 and 3.56–7.50 mm in 2019, while the breeding varieties ranged from 3.69 to 5.74 mm in 2018 and 3.59–6.09 mm in 2019. For the BM, the landraces ranged from 740 to 3,997 gf cm in 2018 and 611–4,288 gf cm in 2019, while the breeding varieties ranged from 800 to 2,439 gf cm in 2018 and 643–2,169 gf cm in 2019. With respect to culm length, the landraces ranged from 84.3 to 143.0 cm in 2018 and 67.9–155.7 cm in 2019, while the breeding varieties ranged from 64.8–116.6 cm in 2018 and 68.7–118.7 cm in 2019. The minimum value was nearly the same between the landraces and the breeding varieties except for the value of culm length in 2018. However, the maximum value of ODMI was 34% higher in 2018 and 23% higher in 2019. Moreover, the maximum value of BM was 64% higher in 2018 and 98% higher in 2019, and the maximum culm length was 23% higher in 2018 and 31% higher in 2019. A comparison of the variance of the mean phenotypic values of landraces and breeding varieties showed that the variance of each trait was higher in landraces than in breeding varieties in both years (Supplementary Data 1). Broad-sense heritability was in the order of culm length, ODMI and BM, from highest to lowest, in both years (Supplementary Data 2). Furthermore, the characteristics of lodging resistance-associated traits were investigated for each landrace and breeding variety. Figure 2 shows the mean values of the landraces and the breeding varieties. The mean value of landraces for ODMI, BM and culm length were higher than that of breeding varieties for both years (*P* < 0.001). These results showed that the phenotypes for the lodging resistance-associated traits were continuously distributed and varied widely, and that the landraces were more diverse than the breeding varieties and have thicker and stronger culms as well as culm length.

To investigate the relationship between each trait, correlation coefficients were calculated for all varieties and for landraces and breeding varieties separately (Supplementary Fig. 1). For all varieties, BM and culm length were highly positively correlated with ODMI (*r* ≥ 0.7). Comparing landraces and breeding varieties, BM and culm length were more positively correlated with ODMI for landraces in each year. In all cases, the correlation coefficient between BM and culm length was lower than that between the other traits.

**Figure 3:**
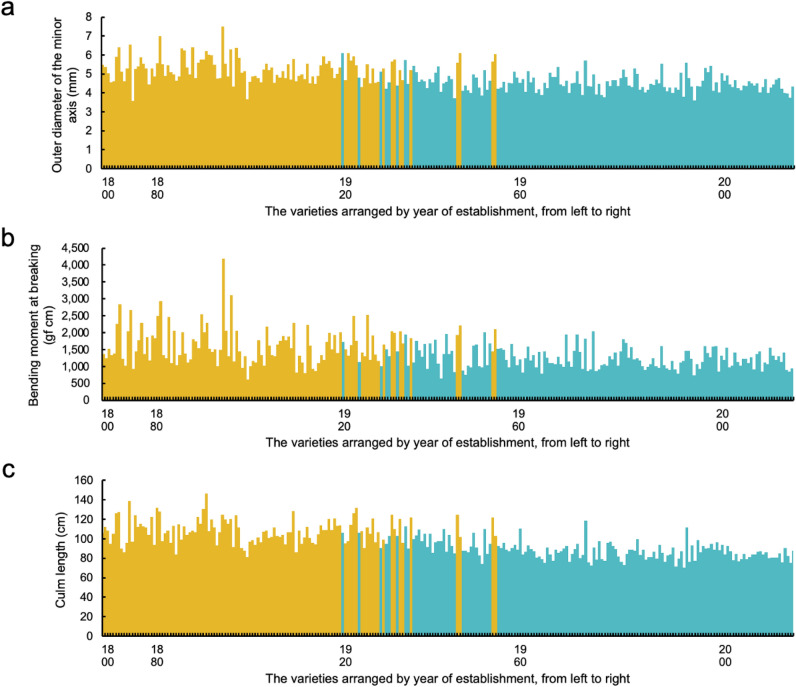
Phenotypic values of the studied varieties arranged in chronological order. Bar graphs of trait values of the varieties arranged by year of establishment, from left to right: (**a**) ODMI; (**b**) BM; (**c**) culm length. The 2019 data of phenotypic values were used, those whose year of establishment was unclear were removed. The yellow and blue bars indicate landraces and breeding varieties, respectively.

The genetic change of rice varieties in Japan in terms of lodging resistance was identified based on their phenotypic values. Figure 3 and Supplementary Fig. 2 shows the phenotypic changes of the varieties arranged in chronological order from the left for each of the lodging resistance-associated traits (data from 2019 were used, those whose year of establishment was unclear were removed). The ODMI and the BM tended to decrease with time. Among the breeding varieties, only Zengokuwase, which was established in 1917, showed thick culms that were greater than 6.00 mm (ODMI), and no breeding varieties with thick culms (greater than 5.00 mm) after the year 2000 were found. For the BM, no varieties with values larger than 2,000 gf cm existed after Nadahikari, which was established in 1977. The culm length tended to decrease with time, and among the varieties released since 1960, only three varieties had long culms (more than 100 cm).

### Identification of QTLs associated with strong culms via GWAS

**Figure 4:**
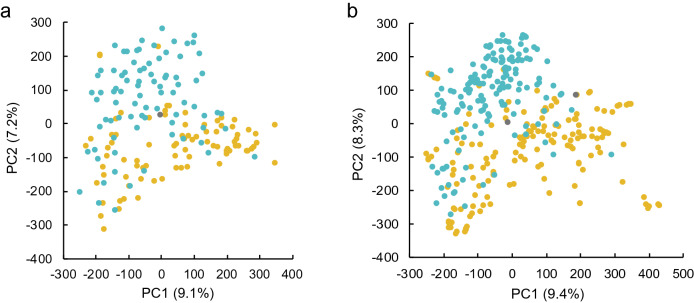
Genetic structure of temperate *japonica* rice varieties in Japan. Principal component analysis of temperate *japonica* rice in Japan based on whole-genome sequence data, with the x-axis representing the PC1 and the y-axis representing the PC2: (**a**) 2018; (**b**) 2019. Values in parentheses indicate the percentage contribution of each principal component. The yellow, blue and black markers indicate landraces, breeding varieties and unidentified varieties, respectively. Principal component analysis was performed using the package ‘pcaMethods’^67^ for R software (https://www.R-project.org/)^65^.

Using GWAS, we tried to identify QTLs containing superior alleles associated with a strong culm present among landraces. It has been reported that false positives are likely to occur if the population structure of the panel used for GWAS is segregated^35^. Therefore, before conducting our GWAS, the genetic structure of the Japanese rice varieties (excluding varieties in Hokkaido) was determined. Figure 4 shows first and second principal component (PC1 and PC2, respectively) from a principal component analysis based on the whole-genome data of the 168 and 326 varieties. These results showed that although there were no clear and strong clusters in the temperate *japonica* tested in this study, there were loose population structures between landraces and breeding varieties in PC1 and PC2. No distinct population structures between landraces and cultivars were found in PC3 and PC4 (Supplementary Fig. 3). Therefore, in addition to the GWAS without principal components (PCs), GWAS with PC1 and GWAS with PC1 and PC2 were also performed.

**Figure 5:**
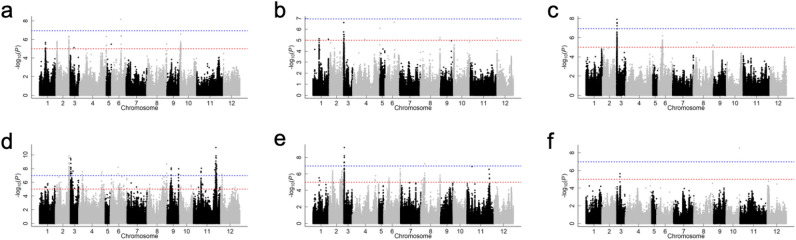
Manhattan plots of lodging resistance-associated traits. (**a**) ODMI in 2018; (**b**) BM in 2018; (**c**) culm length in 2018; (**d**) ODMI in 2019; (**e**) BM in 2019; (**f**) culm length in 2019. The x-axis indicates the SNPs or indels that physically mapped on each chromosome. The red and blue dashed lines indicate the threshold lines (− log_10_ (*P*) = 5) set in this study and the Bonferroni correction, respectively. Manhattan plots were created using the package ‘rrBLUP’^68^ for R software (https://www.R-project.org/)^65^.

Next, GWAS was performed to identify QTLs associated with a strong culm. Figure 5 and Table 1 show Manhattan plots for each trait and the QTLs identified in both years without PCs as fixed factors. Q–Q plots, Local Manhattan plots and linkage disequilibrium (LD) can be found as Supplementary Figs. 4–10. For the ODMI, the same peaks above the threshold (− log_10_ (*P*) > 5) were detected on chromosomes (chrs.) 2S, 2L, 6L, 8L and 10S in both years. With respect to the BM, the same peaks above the threshold (− log_10_ (*P*) > 5) were detected on chr. 3S in both years. Peaks above the threshold (− log_10_ (*P*) > 5) were detected for culm length in each year, but they were not overlapping in both years. The peaks at 1S for ODMI and BM were slightly different from year to year. Peaks at 8L were detected for the ODMI. On the other hand, the peak positions were slightly different for the BM between 2018 and 2019. Among these abovementioned peaks, the peak at 2L in 2019 showed the highest value (− log_10_ (*P*) = 9.89) for the ODMI, and the peak at 3S in 2019 showed the highest value (− log_10_ (*P*) = 9.21) for the BM. A peak at 3S was also detected for ODMI in 2019 and culm length in 2018. When PC1 was considered as a fixed factor, the peaks showed less variation compared to when PCs were not considered, but the peak at 2L was higher for ODMI in 2019 (Fig. 5, Supplementary Fig. 11). When PC1 and PC2 were considered as fixed factors, only the peaks at 2L were above the threshold for ODMI in both years (Fig. 5, Supplementary Fig. 12). The peaks at 3S, which were above the threshold (− log_10_ (*P*) > 5) for ODMI in 2019, for BM in 2018 and 2019 and for culm length in 2018 when PCs were not considered, remained above the threshold (− log_10_ (*P*) > 5), although the peak was reduced in all cases when PC1 and PC2 were considered as fixed factors (Fig. 5, Supplementary Fig. 12). Q–Q plots with PC1 considered and those with PC1 and PC2 considered can be found as Supplementary Figs. 13, 14.

**Table 1:** Positions of peaks identified in both years.

Trait	Chr	Interval (Mb)	Peak maker position in 2018	Peak maker position in 2019	− log_10_ (*P*) in 2018	− log_10_ (*P*) in 2019
Outer diameter of the minor axis	2S	3.8–4.0	3,949,848	3,963,409	5.78	5.62
	2L	28.7–29.3	29,197,385	28,876,750	6.35	9.89
	6L	22.7	22,664,648	22,658,357	6.40	6.81
	8L	26.2–26.8	26,259,466	26,259,466	5.51	7.32
	10S	0.0–0.1	75,142	27,796	5.25	5.46
Bending moment at breaking	3S	0.5–0.9	836,083	920,744	6.61	9.21

### Pleiotropic effects and combined effects of the QTLs

**Figure 6:**
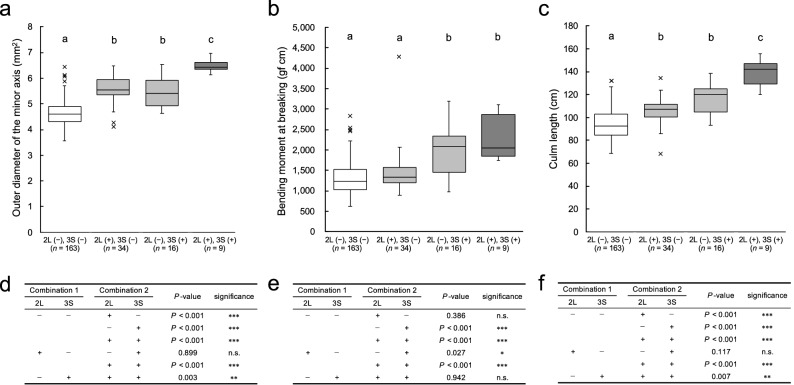
Combined effects of QTLs for lodging resistance-associated traits. Box plots of the trait values by the genotypes classified at the peak marker position in 2019: (**a**) ODMI; (**b**) BM; (**c**) culm length. Test results: (**d**) ODMI; (**e**) BM; **f** culm length. “2L” and “3S” mean the peak positions on chrs. 2L and 3S, respectively. “(−)” and “(+)” indicate the reference and alternative genotypes, respectively (based on the Nipponbare genome). The white, grey and black boxplots indicate the genotype combinations where both are references, one is a reference and the other is an alternative, and both alternatives, respectively. In the boxplots, different letters indicate significant differences at *P* < 0.05 (Steel–Dwass’s test). In the table, n.s. indicates no significant difference. *, **, *** indicate significant differences at *P* < 0.05, 0.01 and 0.001, respectively (Steel–Dwass’s test). Steel–Dwass test was performed using the package ‘NSM3’ for R software (https://www.R-project.org/)^65^.

From the results of the GWAS, QTLs with particularly low *P*-values for ODMI or BM were detected on chrs. 2L and 3S, respectively. Therefore, targeting these QTLs, the pleiotropic effects and combined effects associated with lodging resistance were investigated. Figure 6 shows box plots of the trait values by the genotypes classified at the peak marker position in 2019. The genotypes for each variety can be found as Supplementary Data 3. The varieties in which the reference genotype (based on the Nipponbare genome) of the QTLs on chr. 2L was replaced by the alternative genotype had significantly higher ODMI and culm length values than those that was not replaced. In the comparison of breeding varieties only, the mean values of all traits were increased in varieties where the QTL of chr. 2L was replaced by the alternative genotype, but the differences were not significant (Supplementary Fig. 15). The varieties in which the reference genotype of the QTL on chr. 3S was replaced by an alternative genotype were significantly higher in ODMI, BM and culm length than those in which this QTL was not replaced. These results indicated that the substitution of the reference genotype for the alternative genotype of the QTL on chr. 2L had a pleiotropic effect on increasing the ODMI and the culm length, and the substitution of the reference genotype for the alternative genotype of the QTL on chr. 3S had a pleiotropic effect on increasing the ODMI, BM and the culm length. The varieties in which alternative genotypes of the QTLs were substituted on both chrs. 2L and 3S presented ODMI and culm length values that were significantly higher than those of any other variety group, and an increasing combined effect of QTLs on these traits was observed.

**Figure 7:**
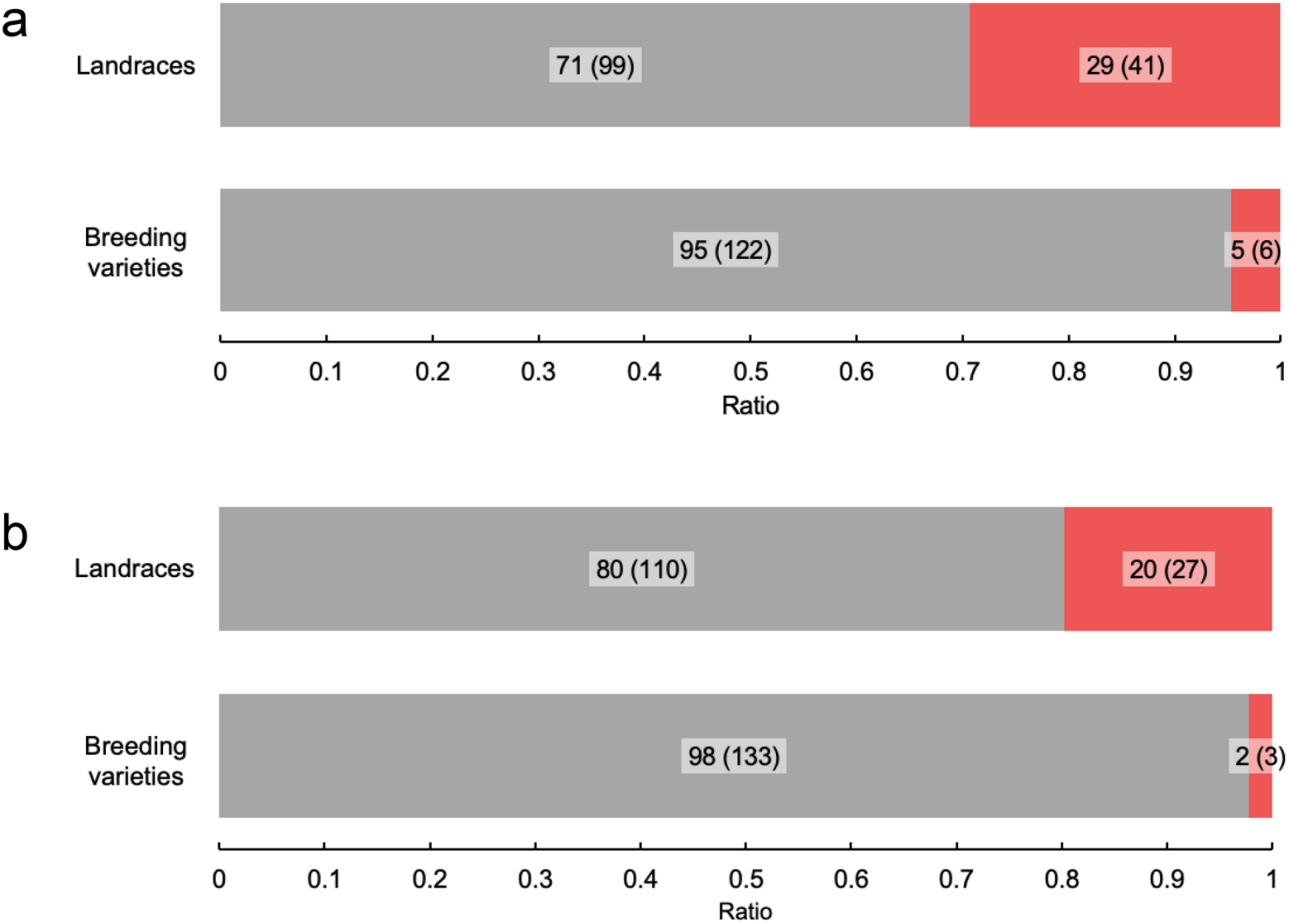
Ratio of genotypes per QTL for landraces and breeding varieties. Stacked bar graphs showing the ratio of genotypes at the peak: (**a**) on chr. 2L; (**b**) on chr. 3S. The grey and red bars indicate the reference and alternative genotypes, respectively. The numbers on the bars indicate the ratio, and the numbers in parentheses indicate the number of varieties.

To investigate the genetic origin of the two QTLs, the genotypes were classified according to peak SNPs for each landrace and breeding variety. Figure 7 shows the ratio of genotypes per QTL for the landraces and breeding varieties. For the QTL on chr. 2L, 29% of the landraces and 5% of the breeding varieties had the alternative genotype with positive effects. For the QTL on chr. 3S, 20% of the landraces and 2% of the breeding varieties had the alternative genotype with positive effects.

### Candidate genes for QTLs associated with strong culms

In LD for the QTL on chr. 2L, there were total of 66 genes according to the MSU Rice Genome Annotation Project (MSU) database and 49 genes according to the RAP-DB (RAP) database. In LD for the QTL on chr. 3S, there were total of 34 genes according to the MSU database and 30 genes according to the RAP database. From these, the candidate genes for each QTL were estimated. Table 2 shows the candidate genes that have mutations in coding DNA sequences (CDSs) and amino acid replacements or deletions in LD for each QTL. For the QTL on chr. 2L, eight candidate genes with amino acid replacements or deletions were evaluated. Among them, *LOC_Os02g47410* was related to protein kinases, *LOC_Os02g47570* was related to phosphatases, and *LOC_Os02g47770* was related to zinc-finger homeodomain proteins. In contrast, *GS2* was included in candidate genes with mutations in the putative promoter region (see Supplementary Table 1). For the QTL on chr. 3S, nine candidate genes with amino acid replacements were evaluated. Among them, *LOC_Os03g02320* and *LOC_Os03g02410* were kinase-associated genes.

**Table 2:** List of candidate genes with amino acid replacements or deletions.

Chr	Position (bp)	MSU ID	RAP ID	Gene symbol	Annotation
2L	28,939,788–28,947,843	*LOC_Os02g47410*	*Os02g0702500*		Protein kinase, putative, expressed
2L	28,967,587–28,971,989	*LOC_Os02g47440*	*Os02g0702800*		Syntaxin, putative, expressed
2L	29,026,099–29,028,176	*LOC_Os02g47510*	*Os02g0704000*	*OsCCD4a*	9-cis-epoxycarotenoid dioxygenase 1, chloroplast precursor, putative, expressed
2L	29,059,400–29,062,130	*LOC_Os02g47560*	*Os02g0704300*		DNA-binding protein, putative, expressed
2L	29,069,440–29,072,094	*LOC_Os02g47570*	*Os02g0704500*		Phosphatase, putative, expressed
2L	29,206,676–29,208,686	*LOC_Os02g47770*	*Os02g0706600*	*OsZHD7*	ZF-HD protein dimerisation region containing protein, expressed
2L	29,270,114–29,274,643	*LOC_Os02g47770*	*Os02g0708100*	*GAT1*	Class I glutamine amidotransferase, putative, expressed
2L	29,324,774–29,328,372	*LOC_Os02g47940*	*Os02g0709200*		Aminotransferase, classes I and II, domain containing protein, expressed
3S	781,076–781,879	*LOC_Os03g02280*	*Os03g0113900*	*OsS40-4*	DUF584 domain containing protein, putative, expressed
3S	800,033–809,279	*LOC_Os03g02320*	*Os03g0114300*		STE_PAK_Ste20_STLK.3 -STE kinases include homologs to sterile 7, sterile 11 and sterile 20 from yeast, expressed
3S	812,826–814,725	*LOC_Os03g02330*	*Os03g0114400*		AAA-type ATPase family protein, putative, expressed
3S	848,054–849,141	*LOC_Os03g02390*	*Os03g0114900*		Mitochondrial import inner membrane translocase subunit Tim17, putative, expressed
3S	850,769–851,768	*LOC_Os03g02400*	*Os03g0115000*	*OsENODL10*	Plastocyanin-like domain containing protein, putative, expressed
3S	852,383–857,144	*LOC_Os03g02410*	*Os03g0115100*		GHMP kinases ATP-binding protein, putative, expressed
3S	861,320–862,938	*LOC_Os03g02430*	*Os03g0115300*		PPR repeat domain containing protein, putative, expressed
3S	884,706–886,943	*LOC_Os03g02460*	*Os03g0115700*		Retinol dehydrogenase, putative, expressed
3S	906,127–909,697	*LOC_Os03g02480*	*Os03g0116000*		Inner membrane protein, putative, expressed

Genes of unclear function and genes related to transposon were removed.

## Discussion

### Phenotypic characteristics of lodging resistance in Japanese temperate *japonica* rice varieties

To date, few studies have evaluated the traits associated with strong culms of landraces^13, 28^. However, in this study, by comprehensively analysing the lodging resistance of a large number of varieties, we found that some landraces showed very high culm strength (Fig. 1). According to previous reports, the BM of Takanari, a semi-dwarf strong-culm *indica* and temperate *japonica* cross variety, was approximately 1,276–2,266 gf cm^7, 24, 27^, while that of its sister variety Habataki was approximately 1,650 gf cm^17^. In the present study, among the landrace varieties in Japan, there were varieties with a BM of approximately 4,000 gf cm (Fig. 1). Therefore, it was thought that some semi-dwarf varieties had high lodging resistance and that landraces in Japan had low lodging resistance because they are generally too tall and the effect of strong culms is not enough to counteract the bending moment given by a high centre of gravity, but it was found that some landraces have phenotypic characteristics associated with extremely strong culms. Although stronger culms are required to prevent the long culm plants from lodging, the effect on increasing lodging resistance given by improved strength from the landraces have a potential to be superior than the effect of plant height on decreasing lodging resistance.

### Transition of breeding for lodging resistance in Japanese rice varieties

It has been reported that rice breeding for lodging resistance in Asia has been performed by shortening culms^4, 37^. In this study, it was shown that, compared with the landraces, the breeding varieties had shorter culms (Figs. 1, 2). Furthermore, it was confirmed that culm length tended to decrease over time (Fig. 3, Supplementary Fig. 2). In contrast, the traits associated with strong culms also tended to decrease over time (Fig. 3, Supplementary Fig. 2), and compared with the landraces, the breeding varieties had thinner and weaker culms (Figs. 1, 2). These results suggest that the improvement of lodging resistance has been dependent on breeding for relatively short culms and that breeding for relatively strong culms has not been carried out.

Both beneficial QTLs for strong culm-associated traits on chr. 2L and 3S were found to be present in a relatively high proportion of landraces (Fig. 7). Previous research showed that haplotypes exhibiting a panicle weight-type phenotype of *SPINDLY* (*OsSPY*; involved in gibberellin signalling)^38^ were present at a higher proportion in landraces than in breeding varieties^39^. Moreover, it has been shown that there is a trade-off between panicle weight and panicle number^17^. From these findings, it was suggested that, during the process of breeding, the emphasis on panicle weight changed to panicle number, and at the same time, the culm strength decreased.

### Genetic population structure and strong culm-related loci in Japanese rice varieties detected via GWAS

The risk of false positives has been pointed out in GWAS when the genetic population structure to be analysed is separated^35^. In the panel of Japanese temperate *japonica* varietiess analyzed in this study, no clear separation of population structures as reported for rice subpopulations^40^ was found, while loose population structures were found between landraces and breeding varieties in PC1 and PC2 (Fig. 4). Therefore, in this study, in addition to GWAS without considering PCs, GWAS was also performed considering up to PC2 to increase the reliability of the detected QTLs.

Since the detected QTLs may change due to changes in expressed genes caused by environmental factors such as meteorological conditions^41, 42^, it is necessary to perform GWAS in multiple years to isolate more general loci. In this study, common peaks were detected at chrs. 2S, 2L, 6L, 8L and 10S for ODMI and at chr. 3S for BM in both 2018 and 2019, among which a particularly high peak was detected at chr. 2L for ODMI and at chr. 3S for BM (Fig. 5, Supplementary Fig. 5–10, Table 1). The peaks at chr. 2L for ODMI and 3S for BM remained above the threshold even when PC1 and PC2 were taken into account (Fig. 5, Supplementary Fig. 12). A QTL for culm diameter on chr. 5L was identified in a previous study on 135 temperate *japonica* varieties from different panels^28^, but was not detected in this study. Conversely, the QTLs on chr. 2L and 3S identified in this study were not detected in that previous study^28^. Unlike that previous study^28^, the panel used in the present study included temperate *japonica* varieties from the rice core collection of Japanese landraces composed by National Agriculture and Food Research Organization (NARO) and landraces from Okinawa, which may have unique genetic variations due to geographical factors. Therefore, it is possible that a novel QTL that was not previously detected was identified in this study. On the other hand, the *APO1* and *FC1* identified in previous studies^16, 17^ were not detected in this study. This suggests that the genes that enhance culm strength are different between temperate *japonica* and other rice subpopulations, such as *indica* and tropical *japonica*, and that the novel QTLs detected in this study may be unique to temperate *japonica*.

### The QTL on the long arm of chromosome 2

As a result of investigating candidate genes of this QTL, eight candidate genes with amino acid replacements or deletions were evaluated (Table 2). Among them, *LOC_Os02g47770* is a gene associated with a zinc-finger homeodomain (ZF-HD) protein^43^. Members of the rice ZF-HD gene family *OsZHD1* and *OsZHD2* have been reported to affect leaf morphogenesis and root meristem activity, respectively^44, 45^. These results suggested that, among the candidate genes, *OsZHD7* might be the gene responsible for modulating culm diameter. On the other hand, *GRAIN SIZE 2* (*GS2*) was included in the genes with mutations in the region presumed to be the promoter (Supplemental Table 1). Since it has been reported that *GS2* affects grain size and weight^46, 47^, it might be possible that *GS2* has pleiotropic effects not only on grains but also on culm morphogenesis.

### The QTL on the short arm of chromosome 3

The interval of QTL on chr. 3S was identified as 0.5–0.9 Mb (Table 1). To date, QTLs for grain eating quality have been reported to be at around the 0.5 Mb mark on the short arm of chromosome 3, where the simple sequence repeat marker RM4108 (synonymous with RM14281) is located^48–50^. These previous studies have reported that Koshihikari, which is a good-tasting variety in Japan, has a beneficial allele related to grain eating quality near this marker, but this allele was not present in varieties such as Moritawase and Nipponbare. However, the haplotypes of the QTL located within 0.5–0.9 Mb associated with BM in this study were similar among Koshihikari, Moritawase, and Nipponbare (Table 1, Supplementary Data 4). This suggests that QTLs for BM and grain eating quality are likely to exist outside of LD. This means that there is a strong possibility that some of the varieties already have both strong culm-related and good-grain eating quality-related alleles at the position of chr. 3S. Therefore, these varieties can be used for breeding to improve lodging resistance without decreasing grain eating quality.

It has been shown that genes such as *APO1* and *FC1* have pleiotropic effects on the traits associated with strong culm and panicle morphogenesis^16, 17^. In this study, each trait was positively correlated, and peaks were detected in this QTL not only for BM but also for ODMI and culm length (Supplementary Fig. 1, 7, Fig. 5). From these results, it is necessary to clarify the pleiotropic and physiological functions of this QTL, including panicle morphogenesis, and to evaluate the usefulness of this QTL other than its effect on culm strength.

As a result of investigating candidate genes of this QTL, nine candidate genes with amino acid replacements were evaluated (Table 2). Among them, *LOC_Os03g02320* is related to serine/threonine protein kinase, and *LOC_Os03g02410* is related to GHMP kinase (Table 2). Serine/threonine protein kinases have been reported to affect plant morphogenesis^51–53^. In addition, it has been suggested that GHMP kinases may be involved in multiple responses stimulated by various plant hormones in *Arabidopsis thaliana*^54^. These results suggest that these candidate genes might be responsible for strong culms. In addition, two variants of SNPs replacing amino acid sequences in the CDS of *LOC_Os03g02320* (Supplementary Data 5) were found by the database to be reference types in the *indica* varieties IR8 and Teqing (https://ricegenome.dna.affrc.go.jp/). Therefore, if this is the responsible gene, the alternative allele might be available for breeding these *indica* varieties to enhance culm strength. However, it has been reported that DNA sequence variation in the promoter region and epigenetic mutations such as DNA methylation can modulate plant traits^55, 56^, thus it is necessary to carefully identify the responsible genes by focusing not only on their coding DNA sequence but also on gene expression levels.

### Combined effects of QTLs on culm thickness

Pyramiding QTLs is an important breeding strategy^57^, and it has been reported that additive effects of pyramiding and combining have been achieved for culm thickness^17, 27^. In the present study, as a result of examining the combined effects of QTLs, an additive combined effect was confirmed for the two identified QTLs (Fig. 6). From these results, it was suggested that culm thickness is a trait that tends to have an additive effect due to the accumulation of QTLs, and this trait may have not yet reached its maximum limit. In other words, it was suggested that additional strong-culm lines can be bred by pyramiding these QTLs and the genes associated with strong culms, such as *APO1*^16^ and *FC1*^17^, both of which have been identified thus far.

The combination of QTLs with the highest value for ODMI comprised alternative (non-Nipponbare) types on both chrs. 2L and 3S, and the combination that showed the lowest value comprised the reference (Nipponbare) type (Fig. 6). The QTLs on chrs. 2L and 3S of Koshihikari, Hitomebore, Hinohikari and Akitakomachi, which are the most widely cultivated varieties in Japan^58^, were all of the reference type (Supplementary Data 3). These varieties have weak to medium lodging resistance (https://ineweb.narcc.affrc.go.jp/index.html), and rice breeding for lodging resistance in Japan has depended on reduced culm length so far (Fig. 3, Supplementary Fig. 2). Therefore, it was suggested that these major Japanese varieties can be improved for lodging resistance by pyramiding the QTLs of these alternative types.

### Prospect of breeding strategy for the “Next Green Revolution”

In this study, the landrace Houmanshindenine had an ODMI of more than 6.25 mm and a BM of approximately 4,000 gf cm (Fig. 1 and Supplementary Fig. 16). In addition, the genotype of the peak at chr. 2 of Houmanshindenine was an alternative type (Supplementary Data 3), suggesting that it may have a superior allele for strong culm-associated traits. For these reasons, the presence of beneficial alleles that enhance culm strength in landraces has been unutilised for many years; thus, these landraces have not been used as breeding materials and have been rarely inherited in modern breeding varieties.

To achieve high rice grain yields that contribute to food security and constitute the “Next Green Revolution”, one key strategy is to breed varieties with culms strong enough to withstand typhoons of increasing intensity, in addition to the strategy of semi-dwarf. A further increase in lodging resistance by increasing culm strength would also make it possible to obtain next generation plant type with larger above-ground biomass and panicles. The use of superior alleles of these landraces as breeding materials to dramatically increase fertility in the future may help to achieve this strategy.

## Conclusion

In this study, we conducted a comprehensive phenotypic analysis of strong culm-associated traits in temperate *japonica* varieties in Japan. As a result, it was suggested that the improvement of lodging resistance in temperate *japonica* varieties in Japan by breeding was dependent of shortening culms. Furthermore, novel QTLs for culm strength-associated traits were identified using GWAS, and it was suggested that these superior haplotypes mainly exist in landraces. In other words, we have shown that the use of superior strong culm-related genes present in landraces that were previously thought to have low lodging resistance due to their long culm could further improve the lodging resistance of modern breeding varieties.

## Methods

### Plant material and cultivation

Landrace and breeding varieties collected from various regions in Japan except Hokkaido were used for rice (*Oryza sativa* L.) panels. A total of 168 varieties were grown in 2018, and 326 were grown in 2019. The entire list of varieties can be found as Supplementary Data 6. Some of these varieties had the same variety name but were registered under different IDs, and these were tested as different varieties. The realized additive relation matrix was calculated to determine the kinship between the varieties, and the data can be found in Supplementary Data 7 (in 2019; variety numbers are the same as in Supplementary Data 6). All plant seeds used in the current study are owned by Nagoya University and these seeds were obtained under a collaboration agreement with Nagoya University. Experimental research and field studies on plants, including the collection of plant materials in the current study, was carried out in compliance with relevant institutional, national and international guidelines and legislation. Field experiments were carried out in a paddy field in the Field Museum Honmachi, Field Science Center, Faculty of Agriculture, Tokyo University of Agriculture and Technology. The seeds were sown in germination boxes on May 7, 2018, and May 7, 2019. The seedlings at the four-leaf stage were transplanted to a paddy field on May 24, 2018, and May 23, 2019, with one seedling per hill. The planting density was 22.2 hills m^−2^, and the spacing was 15 × 30 cm. The residue of rice from the previous year was ploughed as compost during the winter, and N, P_2_O_5_ and K_2_O were applied at 50 kg ha^−1^, 60 kg ha^−1^ and 60 kg ha^−1^, respectively. Weed and pest control were performed as needed, and the fields were cultivated under irrigated conditions.

### Phenotyping

Approximately 15 days after heading, the main culm of eight and three moderately growing individuals (dough stage) were used for phenotypic measurements in 2018 and 2019, respectively. The arithmetic mean of the traits of this individual replication for each variety was determined as the phenotypic value of the variety for each year. The leaf sheaths wrapped at the basal elongated internodes (the elongated internodes were defined as internodes that extended 4 cm or more) were removed. The ODMI at the centre of the basal elongated nodes was measured using a digital calliper. The BM was measured by a three-point bending test using a Tensilon universal material testing machine (RTG-1210; A&D, Tokyo, Japan) according an established method previously reported^13^. The basal elongated internodes were placed on fulcrums 4 cm apart, and a load was applied to the centre of the internodes and was measured at the time of breaking. BM was calculated by the following Eq. (1):

1 $$\begin{array}{*{20}c} {{\it\text{M}}{\text{ = }}\cfrac{{{\it\text{PL}}}}{{4}}} \\ \end{array}$$

where *M* is the BM, *P* is the load at breaking and *L* is the distance between fulcrums. The culm length was defined as the length of the main culm from the base to the neck of panicle in the stretched state, measured using a ruler. Cross sections of basal elongated internodes were stained with toluidine blue and photographed using a stereoscopic microscope (SZX12; Olympus, Tokyo, Japan).

### Genotyping

DNA extraction and sequencing were performed as described in a previous study^35^. Total DNA was extracted from leaves per variety using the DNeasy Plant Mini Kit (Qiagen, Hilden, Germany). Total DNA was sheared into −500 bp fragments by Covaris S2 (Covaris, Massachusetts, USA) and a DNA library was constructed using the NEBNext DNA Library Prep Reagent Set for Illumina (New England Biolabs, Massachusetts, USA). The DNA library was sequenced by the Illumina HiSeq 2000 (Illumina, California, USA). All reads were mapped to the Os-Nipponbare-Reference-IRGSP-1.0 pseudomolecules by BWA-MEM with the -M option in BWA software^59^. The mapped reads were realigned using RealignerTargetCreator and indelRealigner in GATK software^60^. The SNPs and indels were labelled using GATK’s UnifiedGenotyper with the -glm BOTH option. Nucleotide variants with a missing rate of > 0.2 and a minor allele frequency of < 0.05 were removed, resulting in the identification of 435,768 SNPs and indels in the 168-variety panel, and 477,640 SNPs and indels in the 326-variety panel. For the classification of polymorphisms, the promoter region was defined as the 2 kb region upstream of the translation start of each gene. The effects of genomic variants on gene function were predicted using SnpEff software (version 4.3 T)^61^. The information on gene location and coding sequence was obtained from Generic Feature Format Version 3 (GFF3) of the MSU Rice Genome Annotation Project (http://rice.plantbiology.msu.edu/)^62^. Annotation information for genes was based on the content within the MSU Rice Genome Annotation Project and the RAP-DB (http://rapdb.dna.affrc.go.jp/)^62, 63^. LD heatmaps were constructed using the package ‘LDheatmap’ (version 0.99–7)^64^ for R software^65^. Genes with CDS variations and amino acid replacements or deletions and genes with promoter variations in the LD of each QTL were estimated as candidate genes (Genes of unclear function and genes related to transposon were removed). Sequence information for *indica* varieties for comparison of a candidate gene was obtained from TASUKE + (https://ricegenome.dna.affrc.go.jp/)^66^.

### GWAS

The genetic structure of the analysed population was estimated using the “nipals” parameter of the package ‘pcaMethods’ (version 1.78.0)^67^ for R software^65^. The GWAS was performed using a linear mixed model (LMM) represented by the following Eq. (2):

2 $$\begin{array}{*{20}c} {y = X\beta + Zu + \it\varepsilon } \\ \end{array}$$

where *y* is the vector of phenotypes, *X* is the matrix of fixed effects containing the nucleotide polymorphism, the grand mean and PCs calculated by genetic relationship analysis, *β* is the vector of effects, *Z* is the incidence matrix relating *y* and *u*. The variable *u* is a model of the genetic background of each variety as a random effect, with *u* ∼ *N*(0, *Kσ*^2^_g_), where *K* is the kinship matrix calculated from the nucleotide polymorphisms and *σ*^2^_g_ is the genetic variance. *ɛ* is the matrix of residual effects, with *ɛ* ∼ *N*(0, *Iσ*^2^_e_), where *I* is the identity matrix and *σ*^2^_e_ is the residual variance. The realized additive relation matrix was computed using a function “A.mat” and GWAS was performed without considering the principal component, with a fixed factor (PC1) and with fixed factors (PC1 and PC2) for the landraces and the breeding varieties using a function “GWAS” in the package ‘rrBLUP’ (version 4.6.1)^68^ for R software^65^. In the present GWAS, the threshold was set at − log_10_ (*P*) > 5, which was higher than that of several previous studies using rice panels^69–71^.

### Statistical analysis

Two-tailed Welch’s t-test was performed by R software (version 4.0.5)^65^ to compare whether there was a difference in the means of phenotypic trait values between the groups of landraces and breeding varieties and between breeding varieties with and without a QTL. Steel–Dwass test was performed using the “pSDCFlig” function with “Asymptotic” method of the package ‘NSM3’ (version 1.16) for R software^65^ to compare the multiple groups classified by genotypes. The correlation coefficients between phenotypic trait values were calculated using R software^65^. The coefficients of determination (*R*^2^) and the adjusted *R*^2^ for the year of variety establishment and phenotypic values were calculated using R software^65^. Broad-sense heritability was calculated by the following Eq. (3)–(6)^72, 73^:

3 $$\begin{array}{*{20}c} {H^{2} = \cfrac{{V_{{\text{g}}} }}{{V_{{\text{g}}} + V_{{\text{e}}} }}} \\ \end{array}$$

4 $$\begin{array}{*{20}c} {V_{{\text{g}}} = \cfrac{{M_{{\text{g}}} - M_{{\text{e}}} }}{{\overline{r}}}} \\ \end{array}$$

5 $$\begin{array}{*{20}c} {V_{{\text{e}}} = M_{{\text{e}}} } \\ \end{array}$$

6 $$\begin{array}{*{20}c} {\overline{r} = \left( {n - 1} \right)^{ - 1} \left( {\mathop \sum \limits_{i = 1}^{n} r_{i} - \cfrac{{\mathop \sum \nolimits_{i = 1}^{n} r_{i}^{2} }}{{\mathop \sum \nolimits_{i = 1}^{n} r_{i} }}} \right)} \\ \end{array}$$

where *H*^2^ is broad-sense heritability, *M*_g_ and *M*_e_ are the mean sums of squares for genotype and residual error, obtained from analysis of variance (ANOVA), *r* is the number of replications per genotype.

## Supplementary Information

Supplementary Information 1.

Supplementary Information 2.

Supplementary Information 3.

Supplementary Information 4.

Supplementary Information 5.

Supplementary Information 6.

Supplementary Information 7.

Supplementary Information 8.

Supplementary Information 9.
