# Supplementary material for: Potential of rice landraces with strong culms as genetic resources for improving lodging resistance against super typhoons
Source: Sci Rep. 2021 Aug 4;11:15780. doi: 10.1038/s41598-021-95268-0 (PMC8339031; doi:10.1038/s41598-021-95268-0)
Supplement: Supplementary file 6 — Supplementary Information 6. [file 41598_2021_95268_MOESM6_ESM.docx]

**Supplementary Table 1 List of candidate genes with mutations in the putative promoter region.**

| **Chr.** | **Position (bp)** | **MSU ID** | **RAP ID** | **Gene symbol** | **Annotation** |
| --- | --- | --- | --- | --- | --- |
| 2L | 28,763,385–28,767,194 | *LOC_Os02g47120* | *Os02g0699400* |  | Region found in RelA/SpoT proteins containing protein, expressed |
| 2L | 28,777,426–28,784,586 | *LOC_Os02g47150* | *Os02g0699700* | *OsTOP2* | DNA topoisomerase 2, putative, expressed |
| 2L | 28,808,920–28,811,960 | *LOC_Os02g47190* | *Os02g0700300* | *OsDLN66* | MYB family transcription factor, putative, expressed |
| 2L | 28,863,274–28,866,997 | *LOC_Os02g47280* | *Os02g0701300* | *GS2* | Growth-regulating factor, putative, expressed |
| 2L | 28,894,065–28,897,418 | *LOC_Os02g47310* | *Os02g0701600* | *OsVTE4* | Cyclopropane-fatty-acyl-phospholipid synthase, putative, expressed |
| 2L | 28,914,744–28,916,631 | *LOC_Os02g47350* | *Os02g0701900* |  | Oxidoreductase, short chain dehydrogenase/reductase family, putative, expressed |
| 2L | 28,917,800–28,920,757 | *LOC_Os02g47360* | *Os02g0702000* | *OSOTP51* | PPR repeat domain containing protein, putative, expressed |
| 2L | 28,921,176–28,921,792 | *LOC_Os02g47370* | *Os02g0702100* |  | Transcription factor TF2, putative, expressed |
| 2L | 28,935,436–28,938,889 | *LOC_Os02g47400* | *Os02g0702400* |  | Pectinacetylesterase domain containing protein, expressed |
| 2L | 29,078,261–29,081,208 | *LOC_Os02g47590* | *Os02g0704800* |  | Ornithine carbamoyltransferase, putative, expressed |
| 2L | 29,135,051–29,141,632 | *LOC_Os02g47670* | *Os02g0705600* | *OsPUB29* | bg55, putative, expressed |
| 2L | 29,199,147–29,204,307 | *LOC_Os02g47760* | *Os02g0706500* | *AAA-ATPASE 5* | AAA-type ATPase family protein, putative, expressed |
| 2L | 29,229,969–29,234,195 | *LOC_Os02g47800* | *Os02g0707100* | *OsMDHAR4* | Monodehydroascorbate reductase, putative, expressed |
| 2L | 29,238,064–29,241,762 | *LOC_Os02g47810* | *Os02g0707200* | *OsDOF11* | Dof zinc finger domain containing protein, putative, expressed |
| 2L | 29,258,565–29,265,497 | *LOC_Os02g47840* | *Os02g0707900* |  | Universal stress protein domain containing protein, putative, expressed |
| 2L | 29,277,662–29,283,858 | *LOC_Os02g47860* | *Os02g0708200* |  | Uridine kinase, putative, expressed |
| 2L | 29,285,009–29,287,551 | *LOC_Os02g47870* | *Os02g0708300* | *OsRBX1a* | Anaphase-promoting complex subunit 11, putative, expressed |
| 2L | 29,287,709–29,291,570 | *LOC_Os02g47880* | *Os02g0708400* |  | Tetratricopeptide repeat domain containing protein, expressed |
| 3S | 726,370–728,004 | *LOC_Os03g02180* | *Os03g0112900* | *OsF5HL2* | Cytochrome P450, putative, expressed |
| 3S | 752,025–756,172 | *LOC_Os03g02240* | *Os03g0113500* |  | AT-GTL1, putative, expressed |
| 3S | 795,385–798,116 | *LOC_Os03g02310* | *Os03g0114200* |  | RNA binding protein, putative, expressed |
| 3S | 842,577–846,408 | *LOC_Os03g02380* | *Os03g0114800* | *OsCd1* | Major facilitator superfamily domain-containing protein 5, putative, expressed |
| 3S | 857,832–859,757 | *LOC_Os03g02420* | *Os03g0115200* |  | KH domain containing protein, putative, expressed |
| 3S | 870,844–875,516 | *LOC_Os03g02440* | *Os03g0115400* | *OsWD40-60* | WD repeat-containing protein 44, putative, expressed |
| 3S | 878,854–881,449 | *LOC_Os03g02450* | *Os03g0115500* |  | Pyridoxamine 5'-phosphate oxidase family protein, putative, expressed |

Genes of unclear function and genes related to transposon were removed.
