# Supplementary material for: Potential of rice landraces with strong culms as genetic resources for improving lodging resistance against super typhoons
Source: Sci Rep. 2021 Aug 4;11:15780. doi: 10.1038/s41598-021-95268-0 (PMC8339031; doi:10.1038/s41598-021-95268-0)
Supplement: Supplementary file 9 — Supplementary Information 9. [file 41598_2021_95268_MOESM9_ESM.pdf]

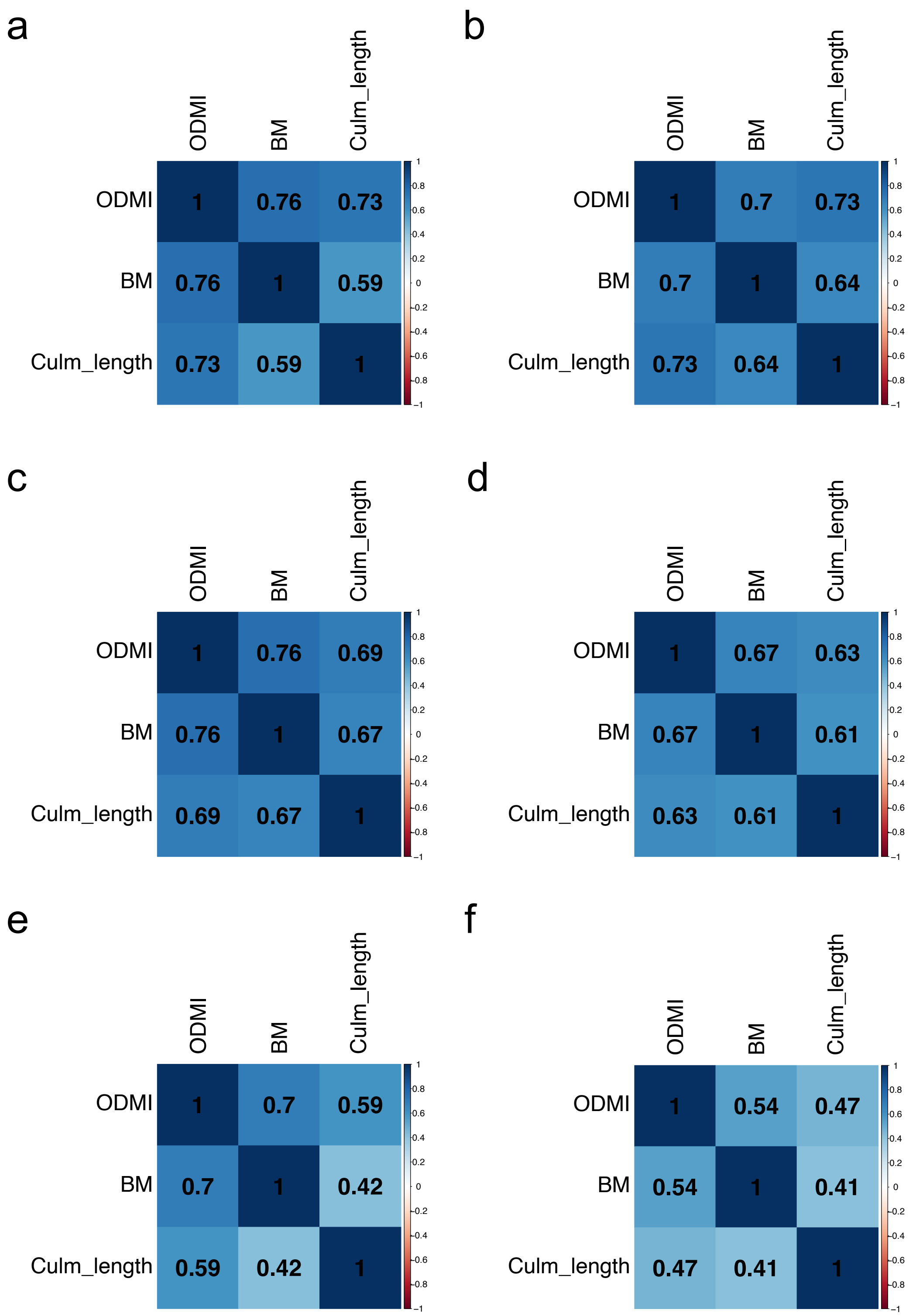

Supplementary Fig. 1: Correlation of phenotypic values for traits associated with lodging resistance. **a** all varieties in 2018; **b** all varieties in 2019; **c** only landraces in 2018; **d** only landraces in 2019; **e** only breeding varieties in 2018; **f** only breeding varieties in 2019. The numbers in the panels indicate the correlation coefficients between phenotypic trait values. The correlation coefficients were calculated using R software (https://www.R-project.org/)[65]. This figure was drawn using the package 'corrplot' for R software[65].

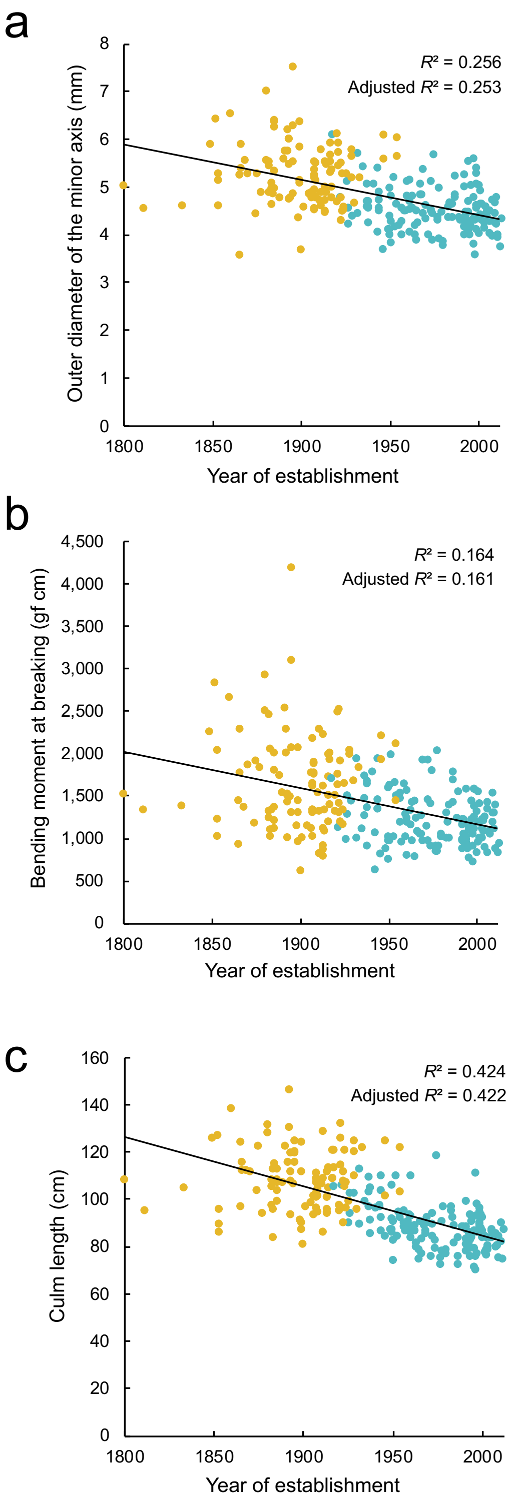

Supplementary Fig. 2: Scatter plots between the year of establishment and the phenotypic values of the varieties.
a ODMI; **b** BM; **c** culm length. The 2019 data of phenotypic values were used, and those with unclear establishment years and those prior to 1800 were removed. The yellow and blue markers indicate landraces and breeding varieties, respectively. The black line indicates linear approximation curve. The R^2 and the adjusted R^2 were calculated using R software (https://www.R-project.org/)[65].

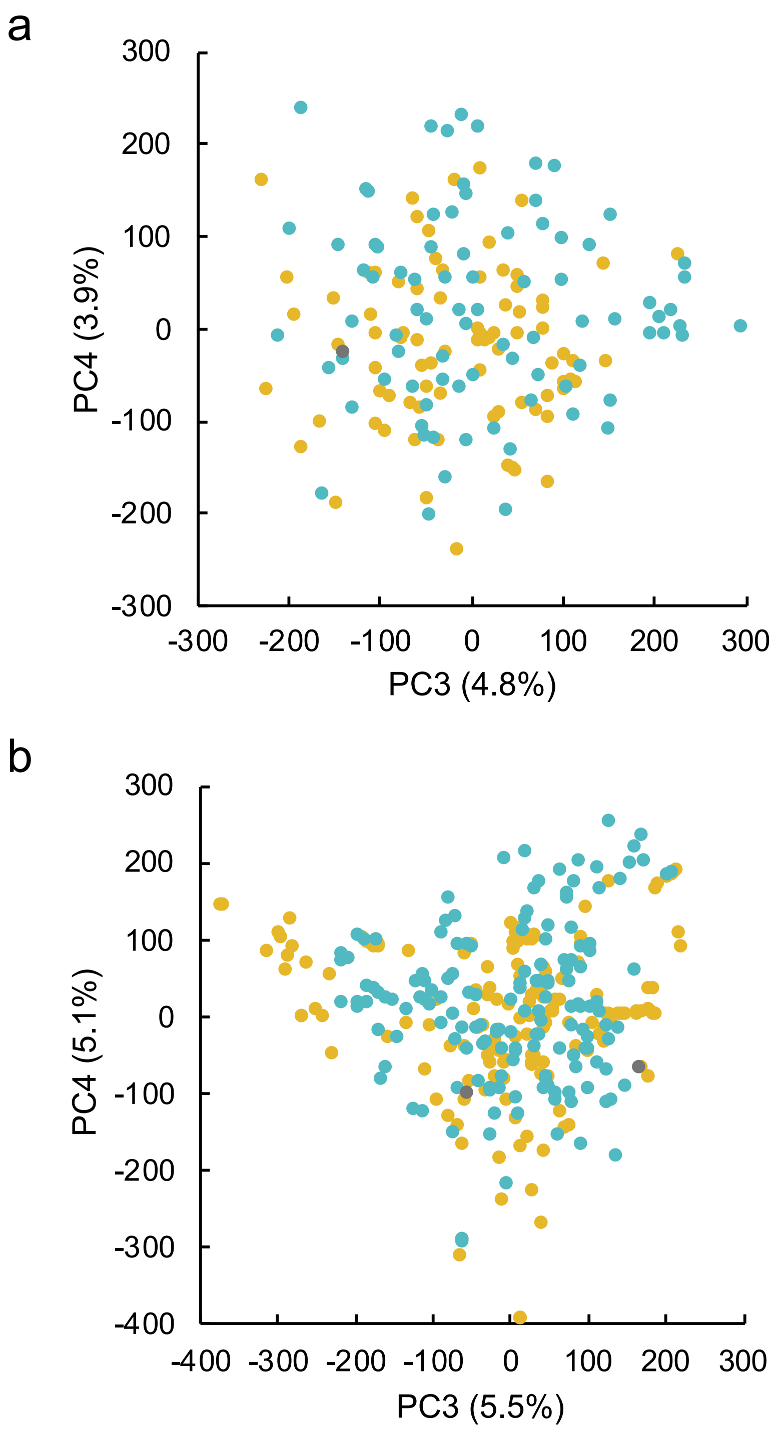

Supplementary Fig. 3: Scatter plots of the third and fourth principal components of temperate *japonica* rice varieties in Japan.
Principal component analysis of temperate *japonica* rice in Japan based on whole-genome sequence data, with the x-axis representing the PC3 and the y-axis representing the PC4: **a** 2018; **b** 2019. Values in parentheses indicate the percentage contribution of each principal component. The yellow, blue and black markers indicate landraces, breeding varieties and unidentified varieties, respectively. Principal component analysis was performed using the package 'pcaMethods'[67] for R software (https://www.R-project.org/)[65].

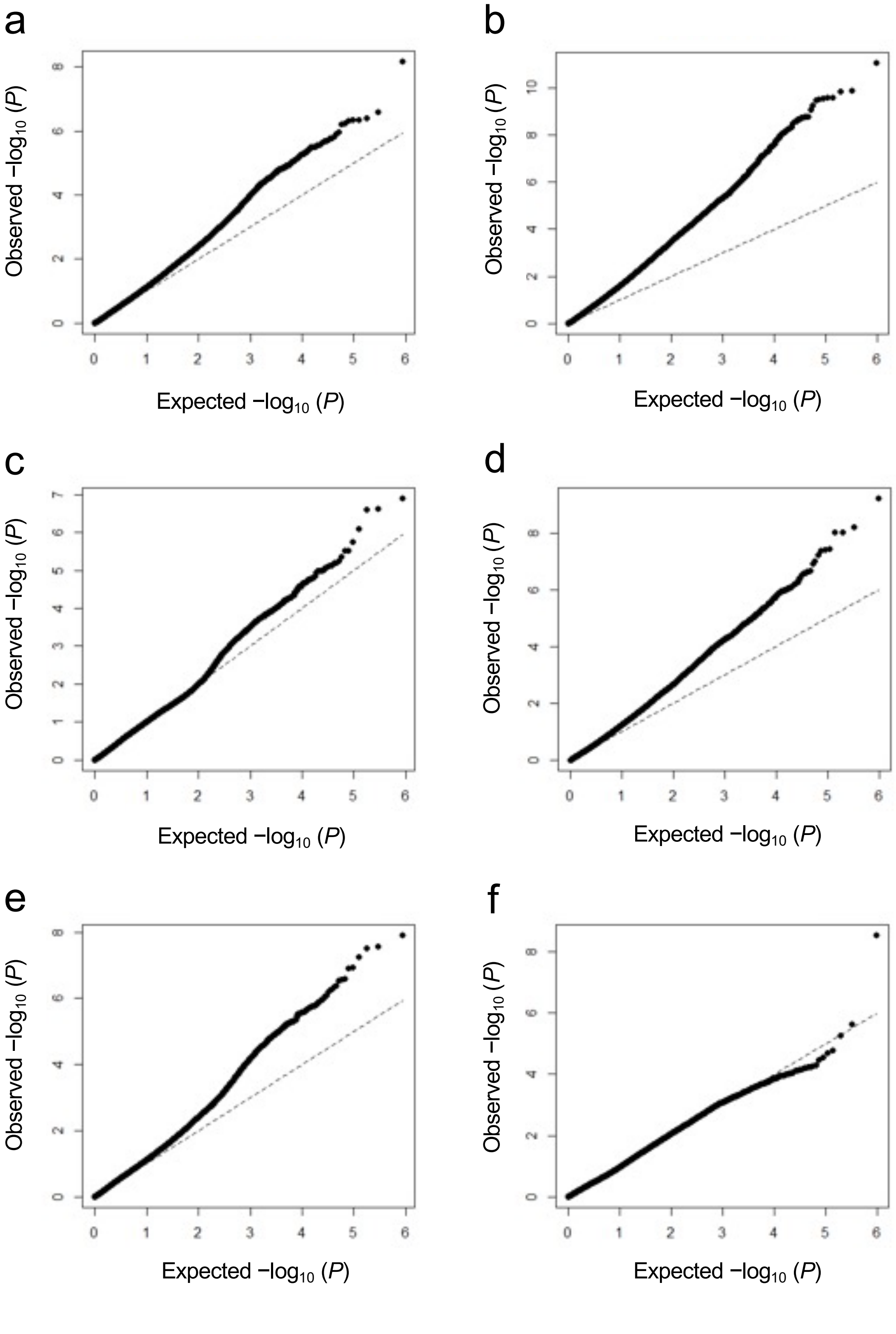

Supplementary Fig. 4: Q–Q plots of GWAS.
a ODMI in 2018; **b** ODMI in 2019; **c** BM in 2018; **d** BM in 2019; **e** culm length in 2018; **f** culm length in 2019. Q–Q plots were created using the package ‘rrBLUP’[68] for R software (https://www.R-project.org/)[65].

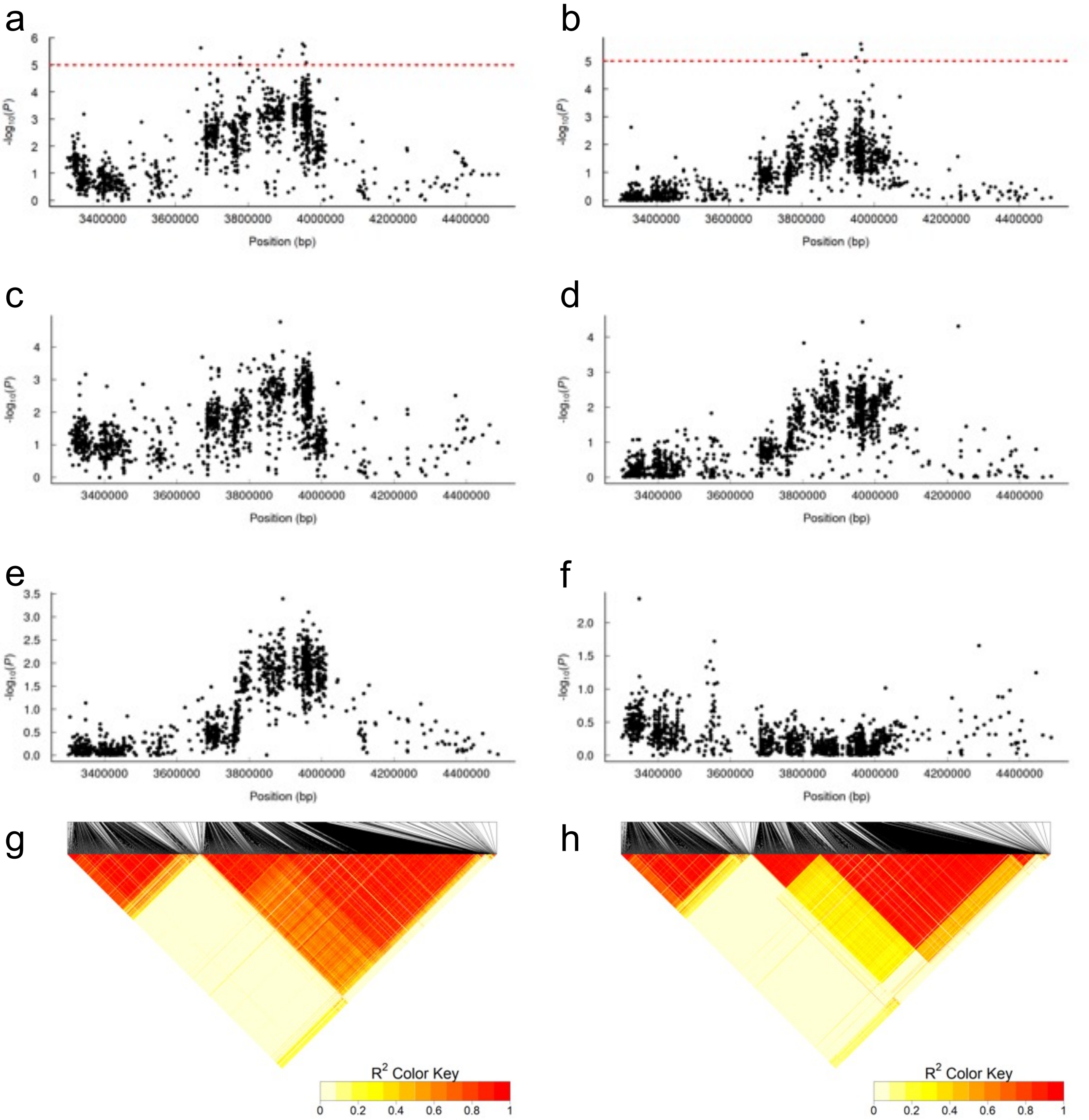

Supplementary Fig. 5: Local Manhattan plots and LD heat maps around the QTL on chr. 2S. Local Manhattan plots: **a** ODMI in 2018; **b** ODMI in 2019; **c** BM in 2018; **d** BM in 2019; **e** culm length in 2018; **f** culm length in 2019. The red dashed lines indicate the threshold lines ($-\log_{10}(P) = 5$) set in this study. LD heat maps: **g** in 2018; **h** in 2019. GWAS was performed using the package 'rrBLUP'[68] for R software (https://www.R-project.org/)[65]. LD heatmaps were constructed using the package 'LDheatmap'[64] for R software[65].

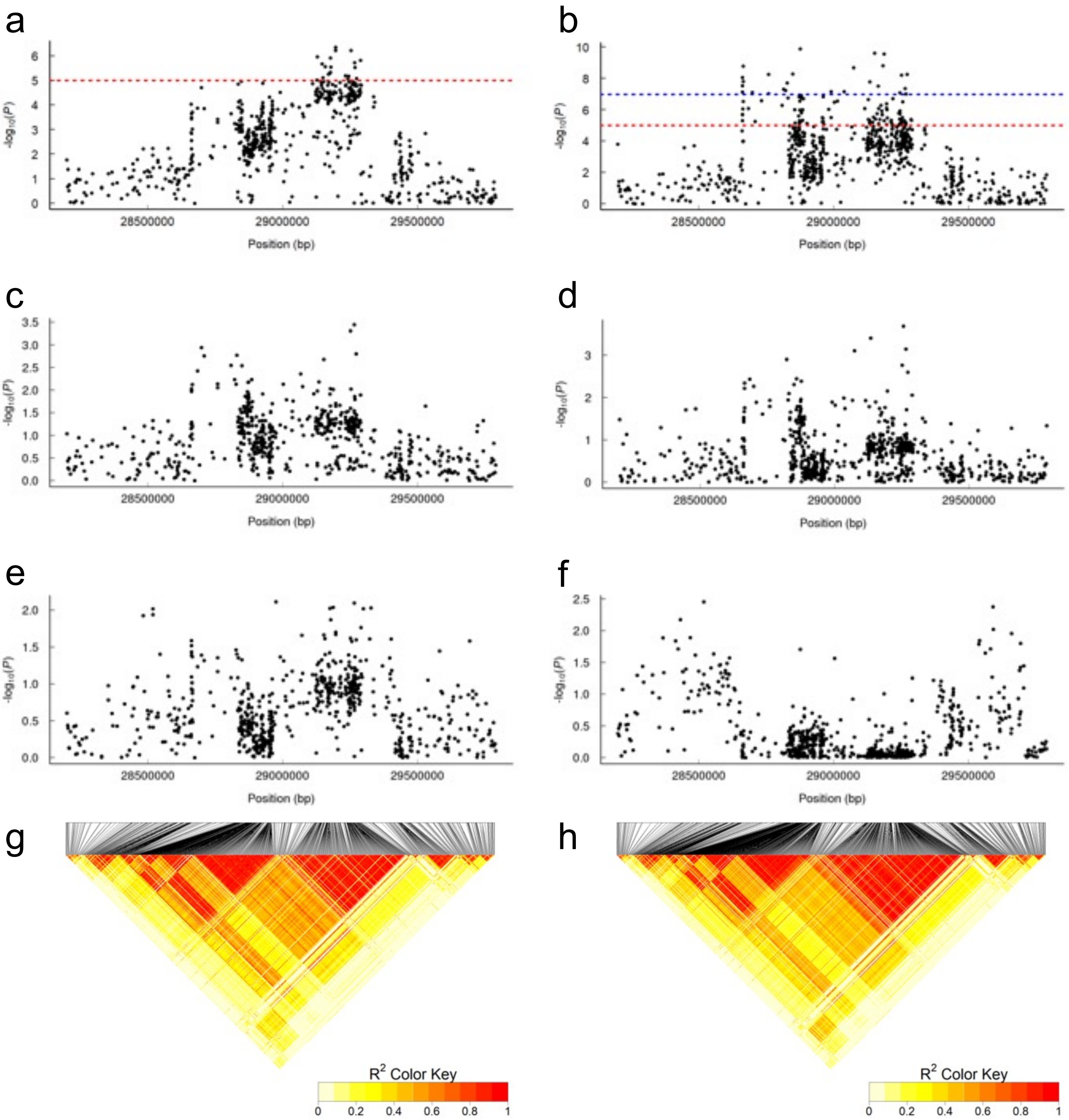

Supplementary Fig. 6: Local Manhattan plots and LD heat maps around the QTL on chr. 2L. Local Manhattan plots: **a** ODMI in 2018; **b** ODMI in 2019; **c** BM in 2018; **d** BM in 2019; **e** culm length in 2018; **f** culm length in 2019. The red and blue dashed lines indicate the threshold lines ($-\log_{10}(P) = 5$) set in this study and the Bonferroni correction, respectively. LD heat maps: **g** in 2018; **h** in 2019. GWAS was performed using the package 'rrBLUP'[68] for R software (https://www.R-project.org/)[65]. LD heatmaps were constructed using the package 'LDheatmap'[64] for R software[65].

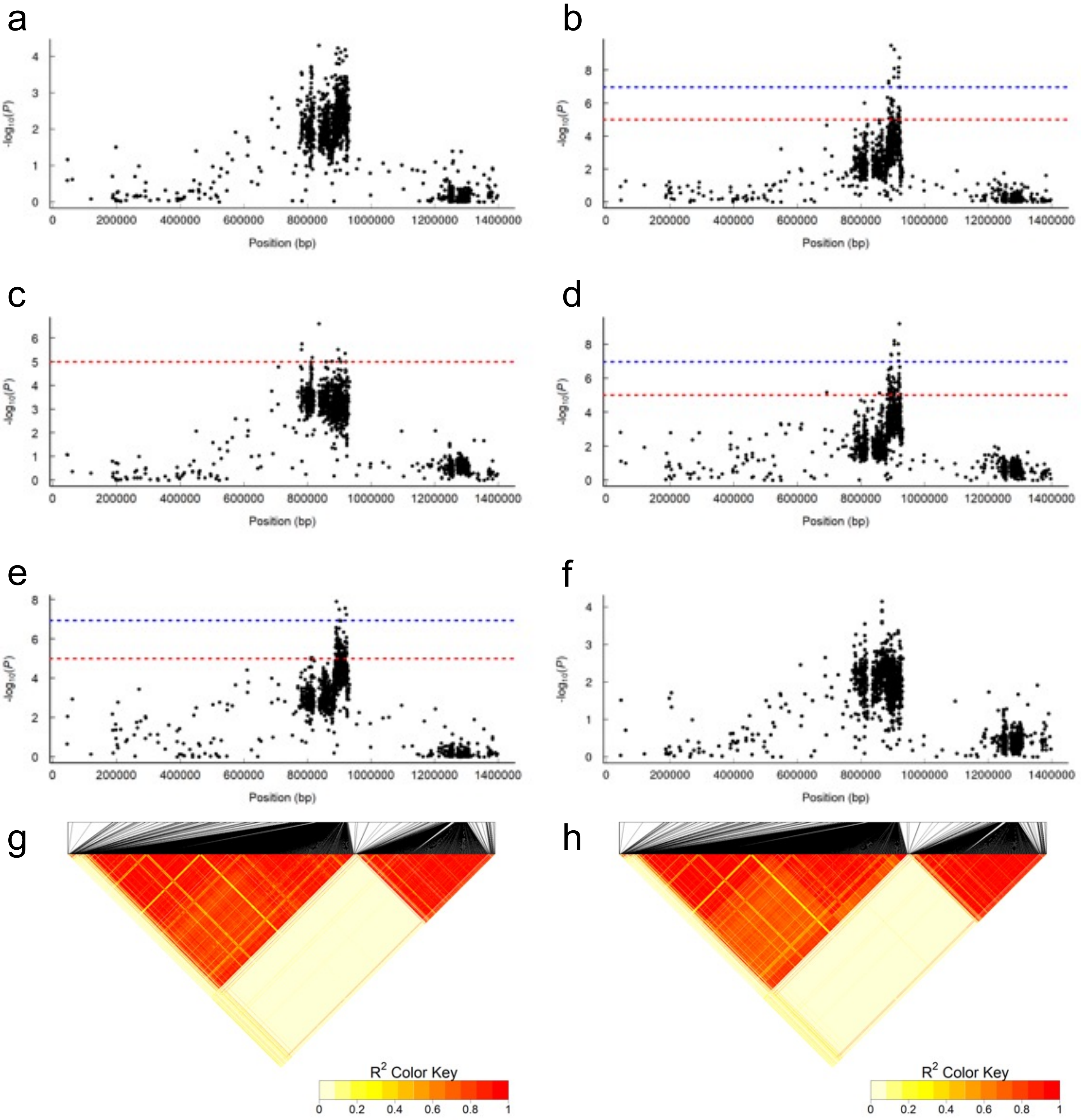

Supplementary Fig. 7: Local Manhattan plots and LD heat maps around the QTL on chr. 3S. Local Manhattan plots: **a** ODMI in 2018; **b** ODMI in 2019; **c** BM in 2018; **d** BM in 2019; **e** culm length in 2018; **f** culm length in 2019. The red and blue dashed lines indicate the threshold lines ($-\log_{10}(P) = 5$) set in this study and the Bonferroni correction, respectively. LD heat maps: **g** in 2018; **h** in 2019. GWAS was performed using the package 'rrBLUP'[68] for R software (https://www.R-project.org/)[65]. LD heatmaps were constructed using the package 'LDheatmap'[64] for R software[65].

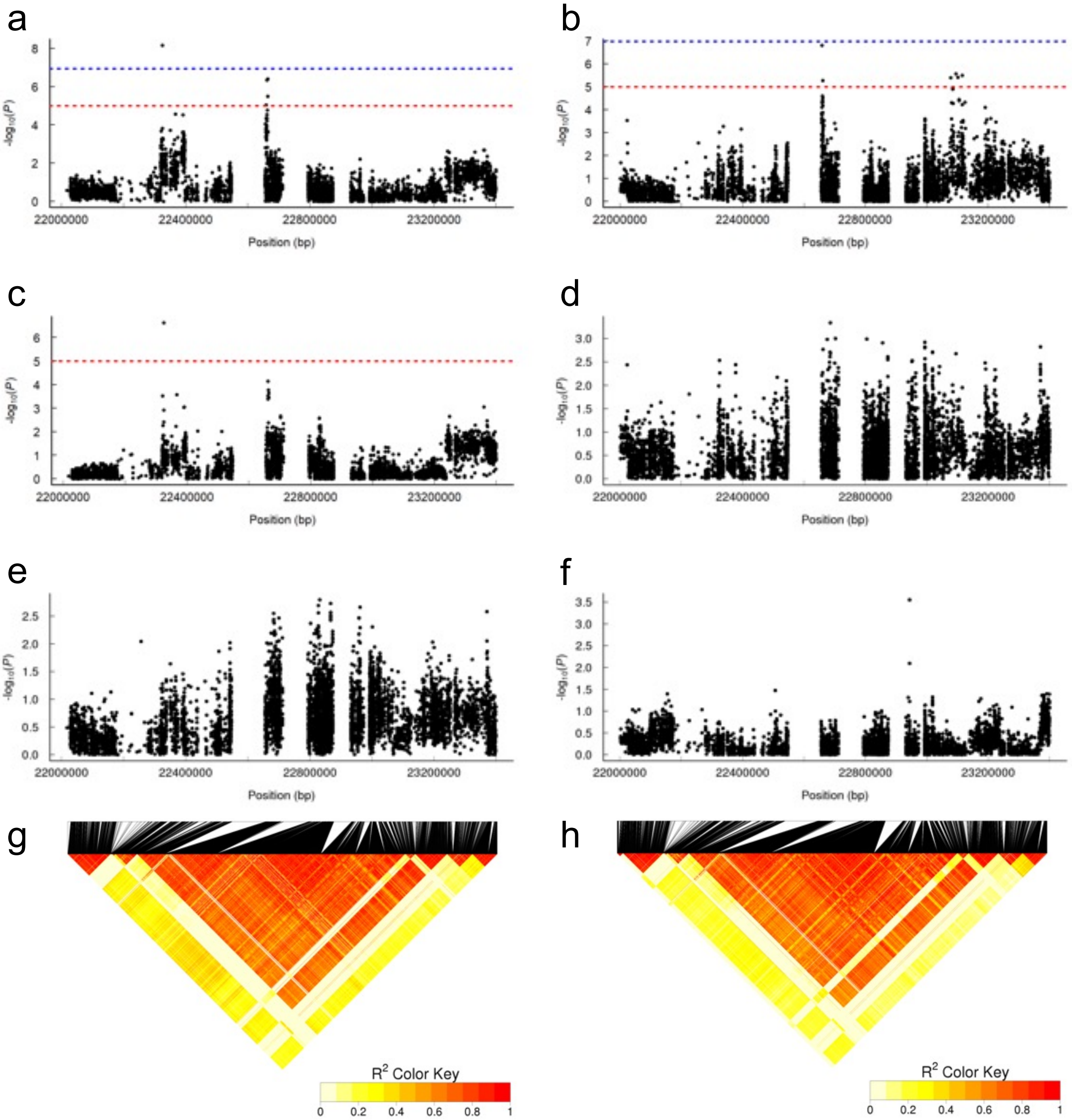

Supplementary Fig. 8: Local Manhattan plots and LD heat maps around the QTL on chr. 6L. Local Manhattan plots: **a** ODMI in 2018; **b** ODMI in 2019; **c** BM in 2018; **d** BM in 2019; **e** culm length in 2018; **f** culm length in 2019. The red and blue dashed lines indicate the threshold lines ($-\log_{10}(P) = 5$) set in this study and the Bonferroni correction, respectively. LD heat maps: **g** in 2018; **h** in 2019. GWAS was performed using the package 'rrBLUP'[68] for R software (https://www.R-project.org/)[65]. LD heatmaps were constructed using the package 'LDheatmap'[64] for R software[65].

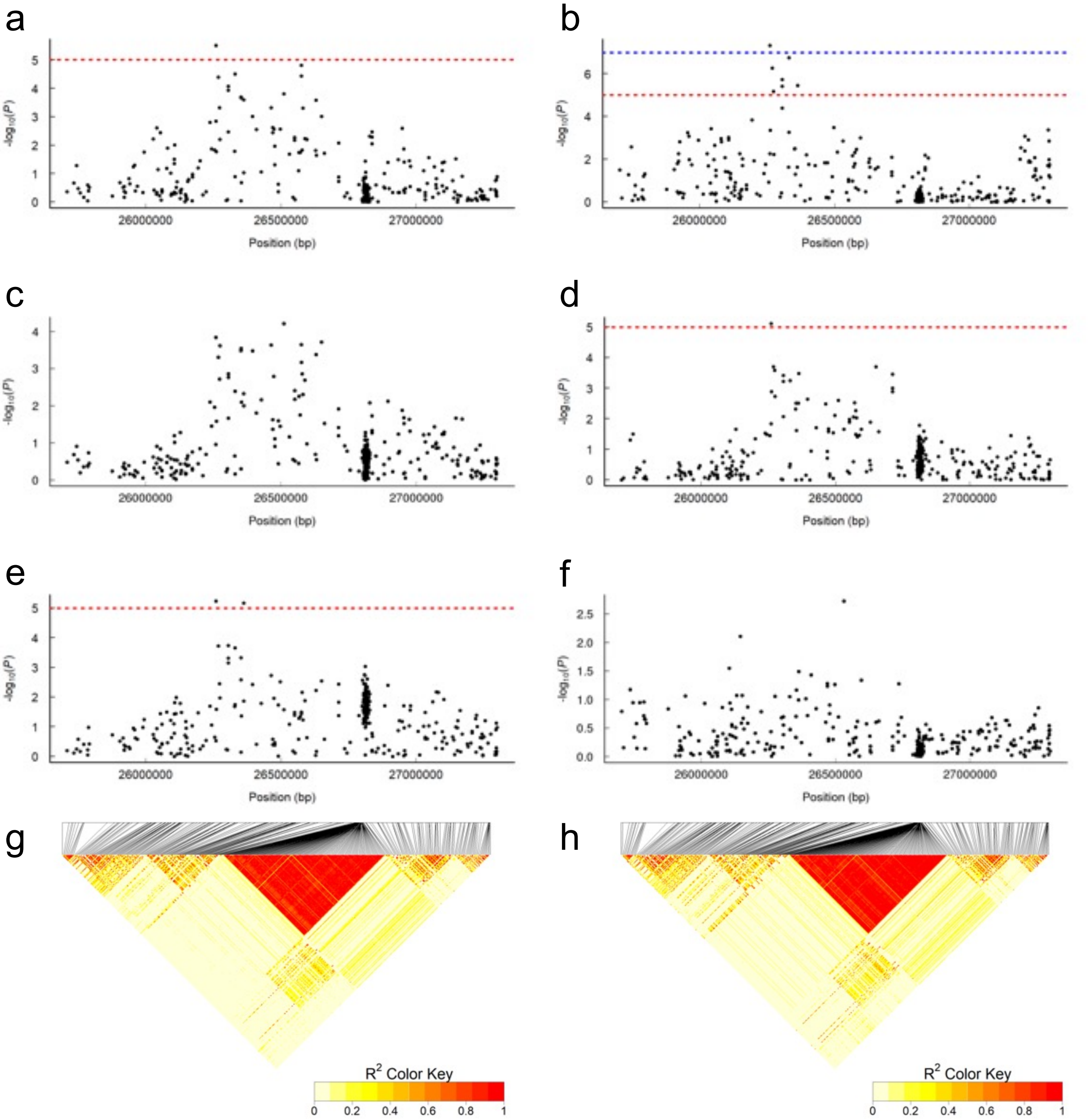

Supplementary Fig. 9: Local Manhattan plots and LD heat maps around the QTL on chr. 8L. Local Manhattan plots: **a** ODMI in 2018; **b** ODMI in 2019; **c** BM in 2018; **d** BM in 2019; **e** culm length in 2018; **f** culm length in 2019. The red and blue dashed lines indicate the threshold lines ($-\log_{10}(P) = 5$) set in this study and the Bonferroni correction, respectively. LD heat maps: **g** in 2018; **h** in 2019. GWAS was performed using the package 'rrBLUP'[68] for R software (https://www.R-project.org/)[65]. LD heatmaps were constructed using the package 'LDheatmap'[64] for R software[65].

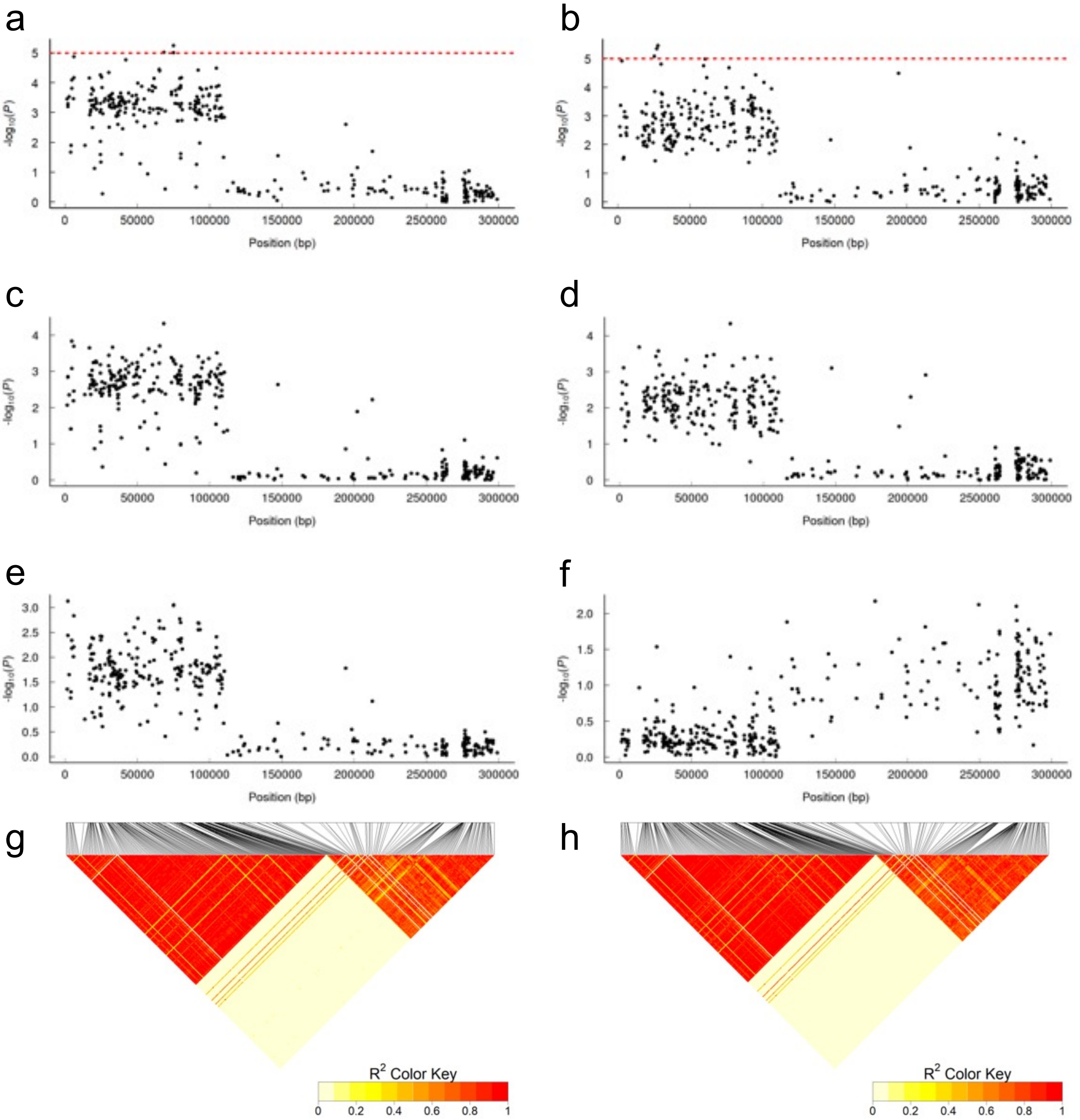

Supplementary Fig. 10: Local Manhattan plots and LD heat maps around the QTL on chr. 10S. Local Manhattan plots: **a** ODMI in 2018; **b** ODMI in 2019; **c** BM in 2018; **d** BM in 2019; **e** culm length in 2018; **f** culm length in 2019. The red dashed lines indicate the threshold lines ($-\log_{10}(P) = 5$) set in this study. LD heat maps: **g** in 2018; **h** in 2019. GWAS was performed using the package 'rrBLUP'[68] for R software (https://www.R-project.org/)[65]. LD heatmaps were constructed using the package 'LDheatmap'[64] for R software[65].

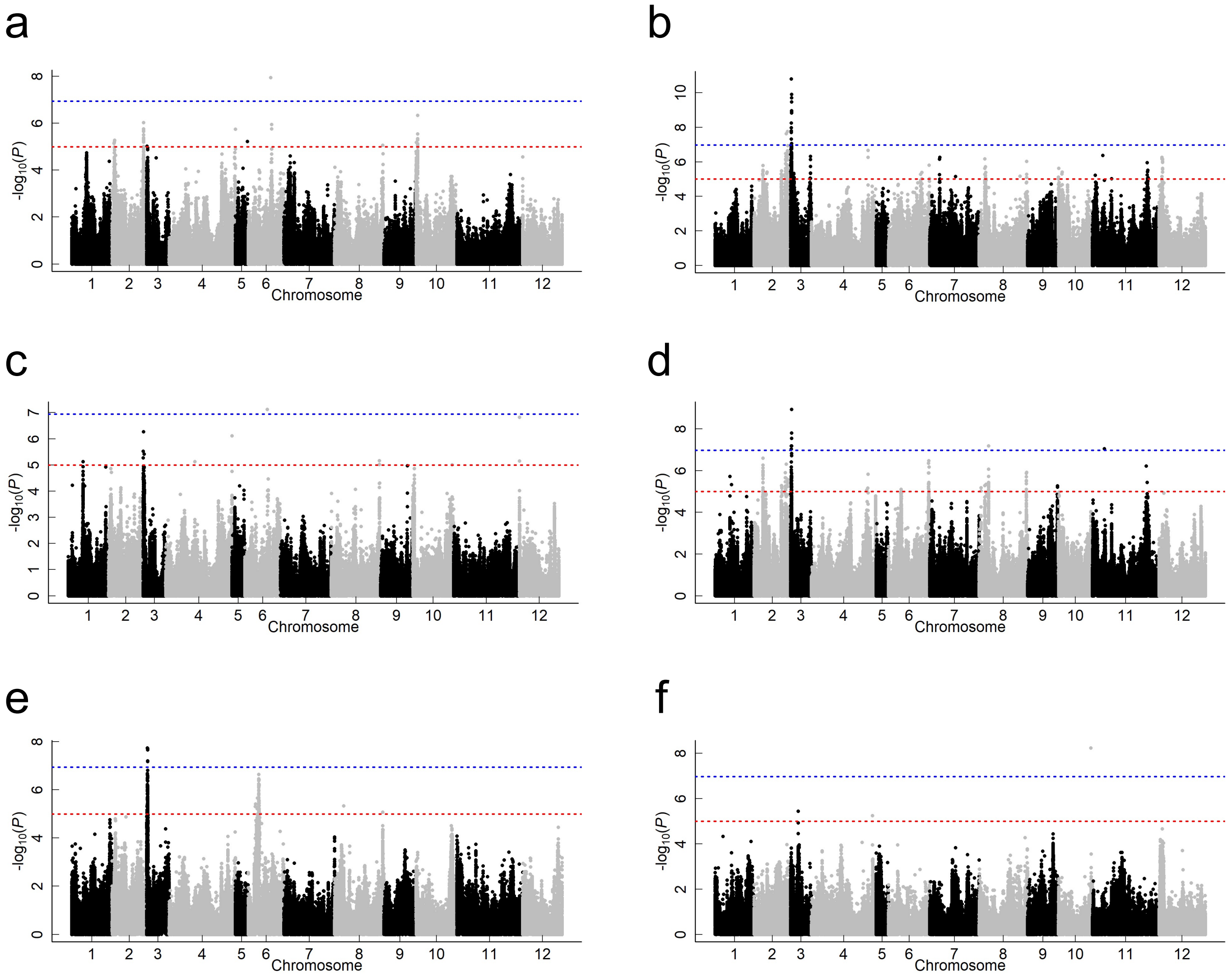

Supplementary Fig. 11: Manhattan plots of the GWAS model with PC1 considered as a fixed effect. **a** ODMI in 2018; **b** ODMI in 2019; **c** BM in 2018; **d** BM in 2019; **e** culm length in 2018; **f** culm length in 2019. The x-axis indicates the SNPs or indels that physically mapped on each chromosome. The red and blue dashed lines indicate the threshold lines ($-\log_{10}(P) = 5$) set in this study and the Bonferroni correction, respectively. Manhattan plots were created using the package 'rrBLUP'[68] for R software (https://www.R-project.org/)[65].

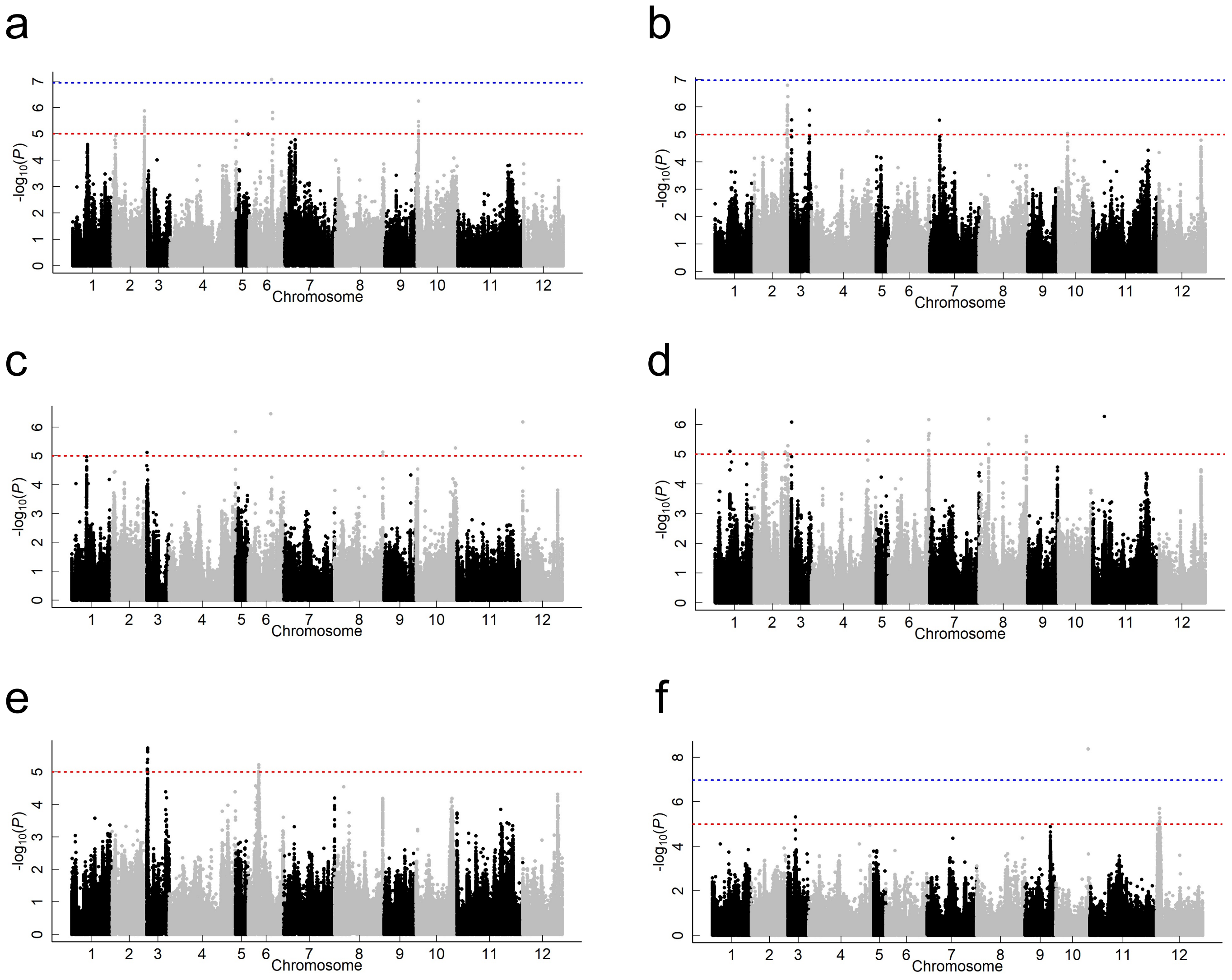

Supplementary Fig. 12: Manhattan plots of the GWAS model with PC1 and PC2 considered as fixed effects.

a ODMI in 2018; **b** ODMI in 2019; **c** BM in 2018; **d** BM in 2019; **e** culm length in 2018; **f** culm length in 2019. The x-axis indicates the SNPs or indels that physically mapped on each chromosome. The red and blue dashed lines indicate the threshold lines ($-\log_{10}(P) = 5$) set in this study and the Bonferroni correction, respectively. Manhattan plots were created using the package 'rrBLUP'[68] for R software (https://www.R-project.org/)[65].

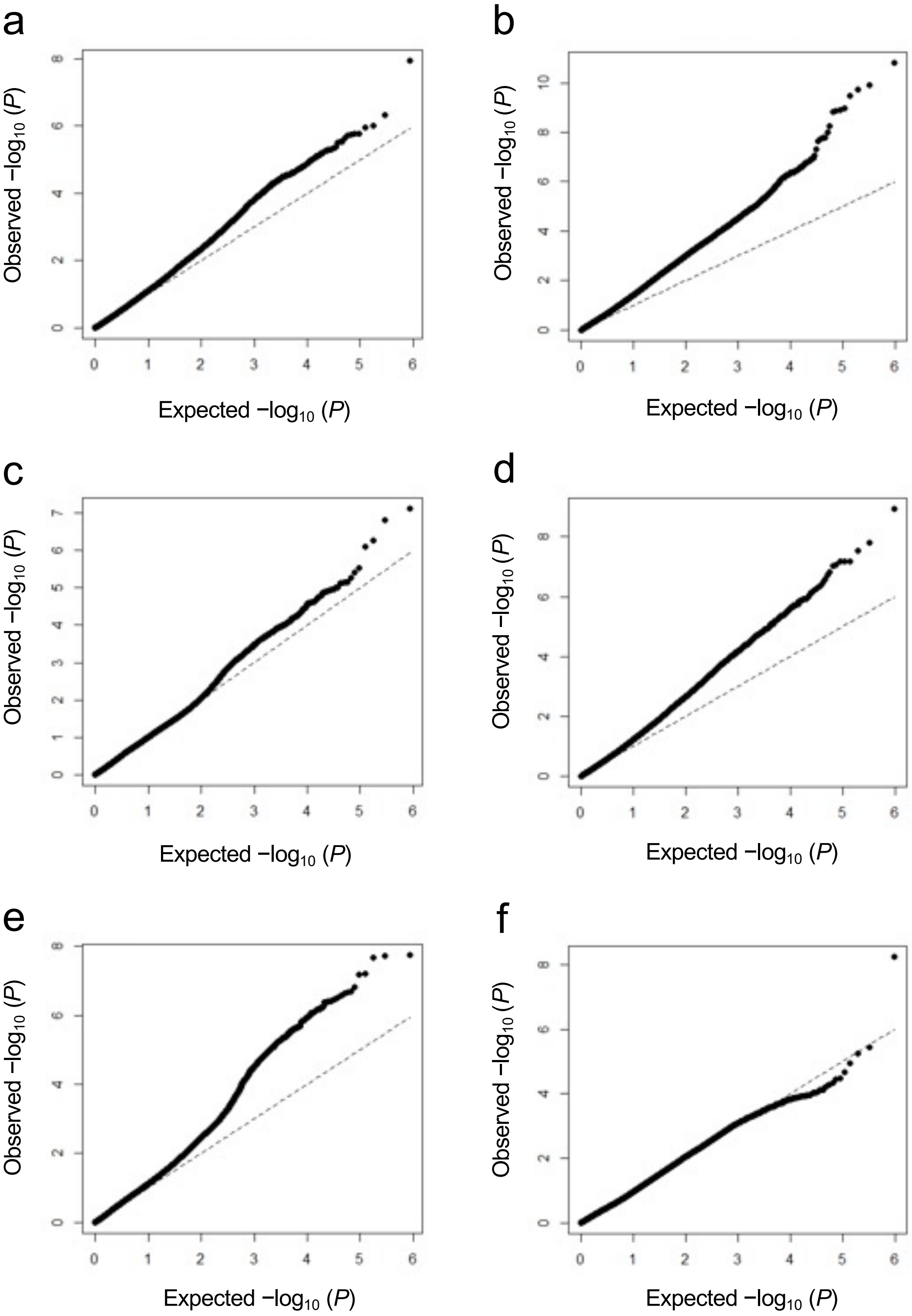

Supplementary Fig. 13: Q–Q plots of GWAS with PC1 considered as a fixed effect.
a ODMI in 2018; **b** ODMI in 2019; **c** BM in 2018; **d** BM in 2019; **e** culm length in 2018; **f** culm length in 2019. Q–Q plots were created using the package 'rrBLUP'[68] for R software (https://www.R-project.org/)[65].

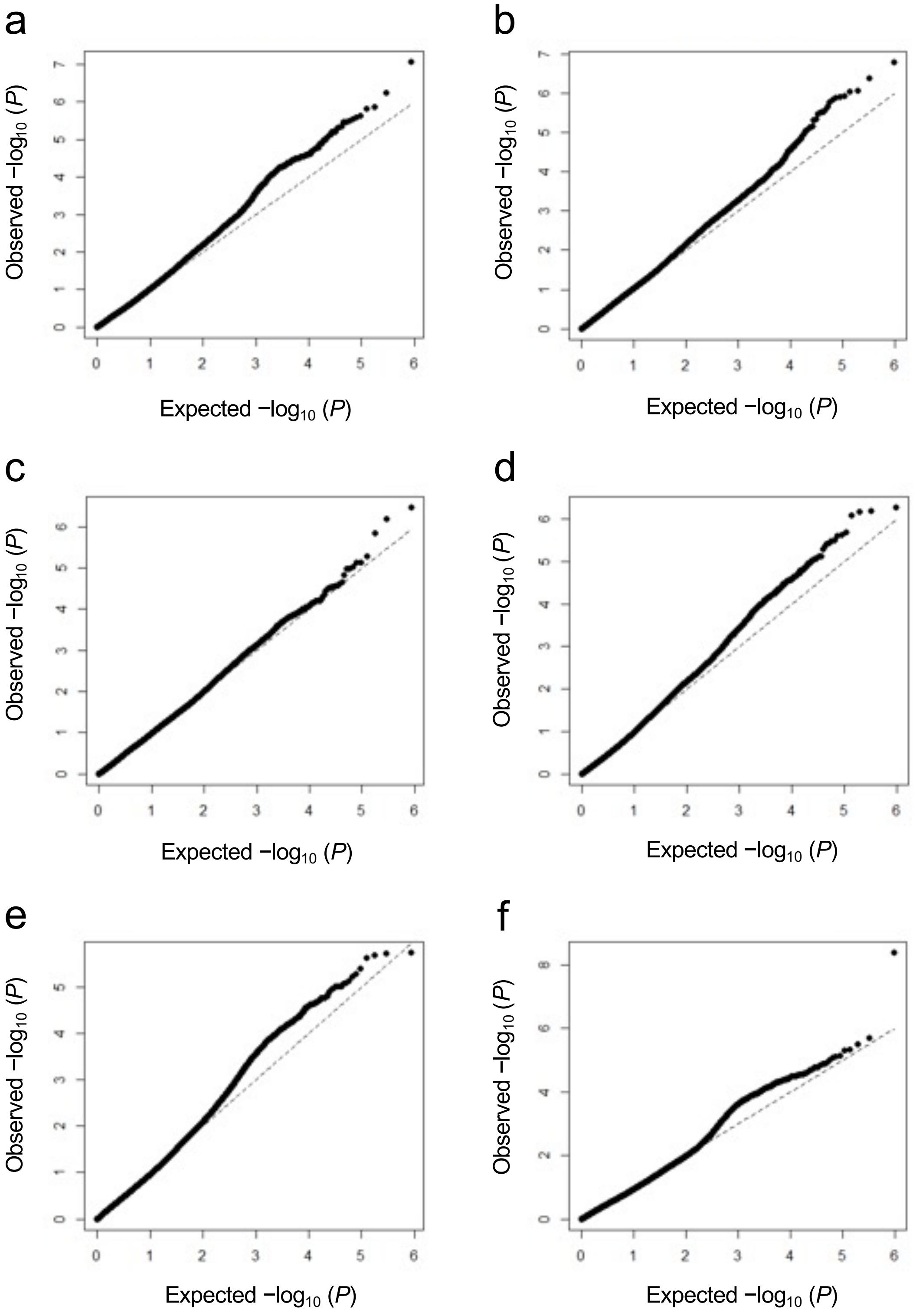

Supplementary Fig. 14: Q–Q plots of GWAS with PC1 and PC2 considered as fixed effects.
a ODMI in 2018; **b** ODMI in 2019; **c** BM in 2018; **d** BM in 2019; **e** culm length in 2018; **f** culm length in 2019. Q–Q plots were created using the package ‘rrBLUP’[68] for R software (https://www.R-project.org/)[65].

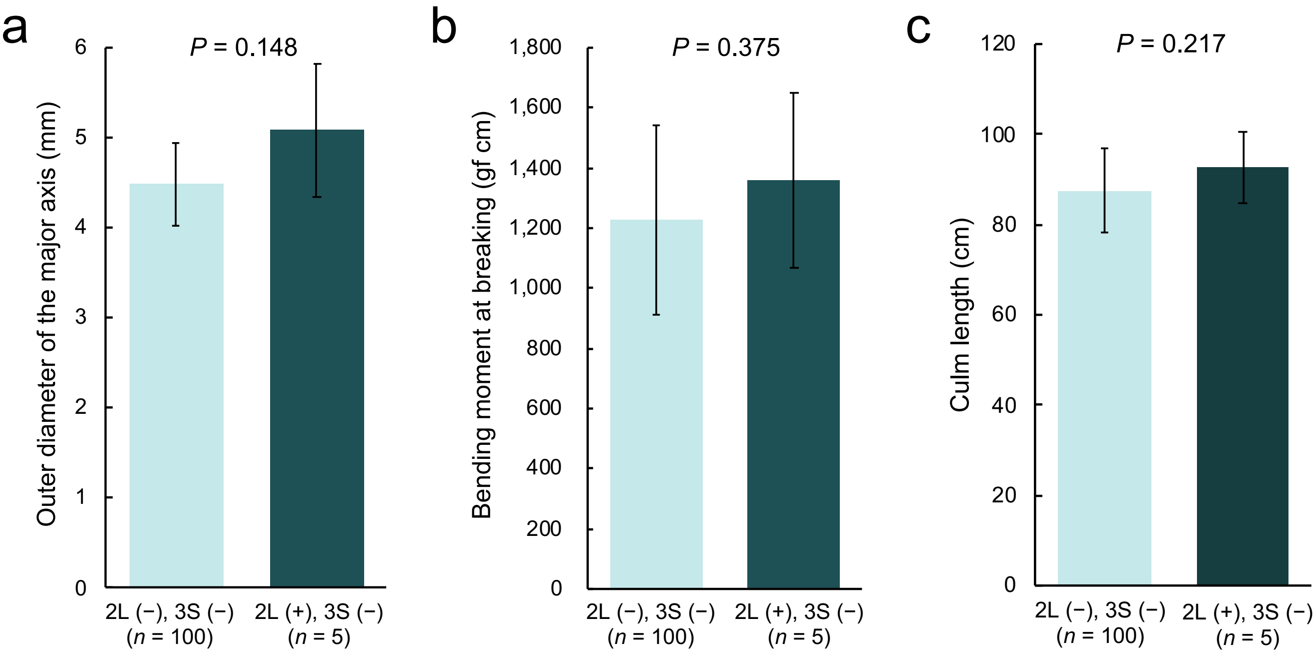

Supplementary Fig. 15: Comparison of trait values between breeding varieties with reference and alternative QTL on chr. 2L.

Bar graphs of mean (SD) trait values for each genotype within the breeding varieties, classified at the peak marker position in 2019: **a** ODMI; **b** BM; **c** culm length. “2L” and “3S” mean the peak positions on chrs. 2L and 3S, respectively. “(−)” and “(+)” indicate the reference and alternative genotypes, respectively (based on the Nipponbare genome). The light and dark blue bars indicate the genotype combinations where both are references, 2L is the alternative and 3S is the reference, respectively. The *P*-values were calculated by two-tailed Welch's t-test, which was performed by R software (https://www.R-project.org/)[65].

a

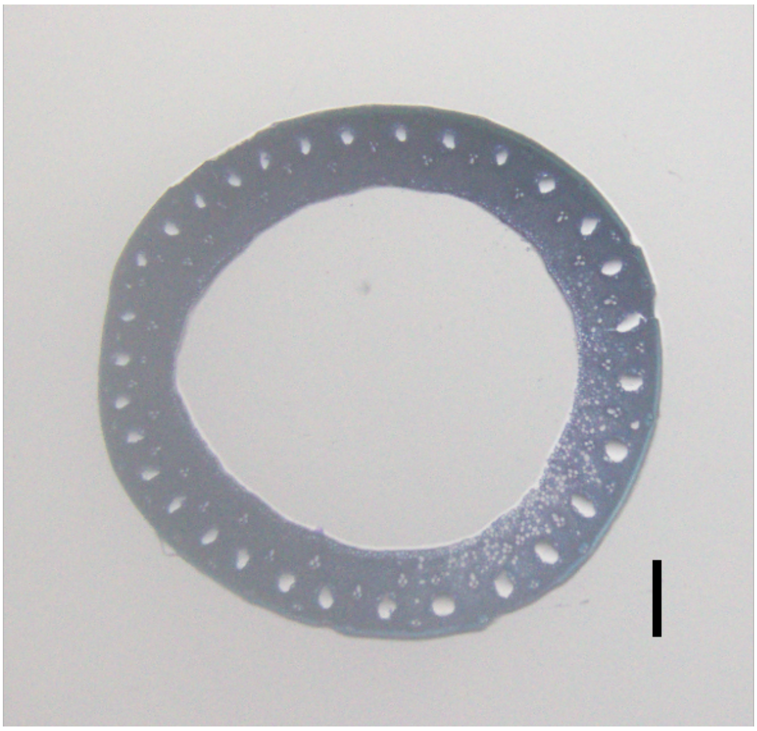

b

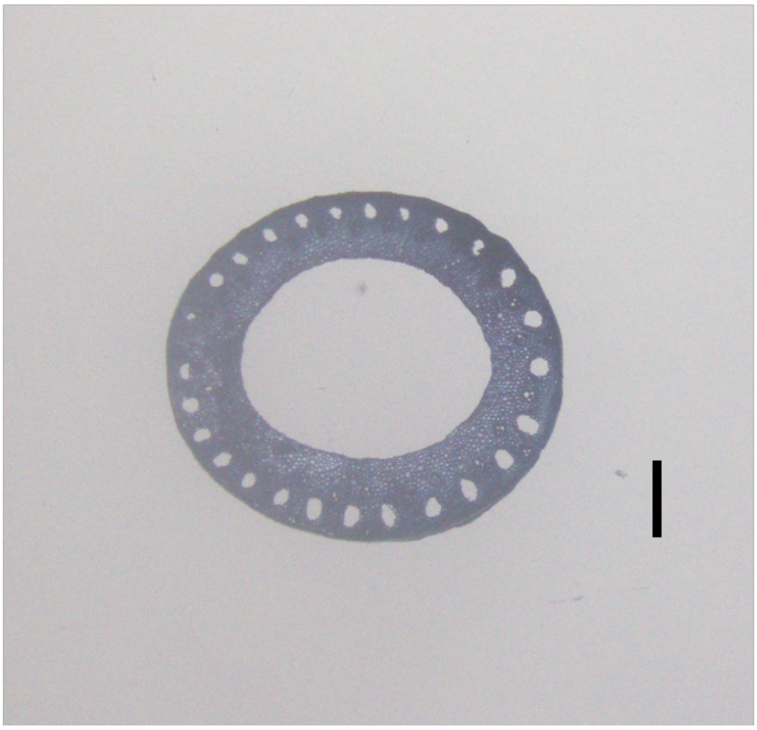

Supplementary Fig. 16: Cross section of basal elongated internodes.
a Houmanshindenine; **b** Koshihikari. Black bar indicates 1 mm.
